# Functional analysis of archaeal MBF1 by complementation studies in yeast

**DOI:** 10.1186/1745-6150-6-18

**Published:** 2011-03-10

**Authors:** Jeannette Marrero Coto, Ann E Ehrenhofer-Murray, Tirso Pons, Bettina Siebers

**Affiliations:** 1 Faculty of Chemistry, Biofilm Centre, Molecular Enzyme Technology and Biochemistry, Biofilm Centre, Faculty of Chemistry, University of Duisburg-Essen, Universitätsstr. 5, (S05 V03 F41), 45141 Essen, Germany; 2 Department for Genetics, University Duisburg-Essen, Universitätsstr. 5, 45117 Essen, Germany; 3 Faculty of Biology, University of Havana, Havana 10400, Cuba

## Abstract

**Background:**

Multiprotein-bridging factor 1 (MBF1) is a transcriptional co-activator that bridges a sequence-specific activator (basic-leucine zipper (bZIP) like proteins (e.g. Gcn4 in yeast) or steroid/nuclear-hormone receptor family (e.g. FTZ-F1 in insect)) and the TATA-box binding protein (TBP) in Eukaryotes. MBF1 is absent in Bacteria, but is well- conserved in Eukaryotes and Archaea and harbors a C-terminal Cro-like Helix Turn Helix (HTH) domain, which is the only highly conserved, classical HTH domain that is vertically inherited in all Eukaryotes and Archaea. The main structural difference between archaeal MBF1 (aMBF1) and eukaryotic MBF1 is the presence of a Zn ribbon motif in aMBF1. In addition MBF1 interacting activators are absent in the archaeal domain. To study the function and therefore the evolutionary conservation of MBF1 and its single domains complementation studies in yeast (*mbf1*Δ) as well as domain swap experiments between aMBF1 and yMbf1 were performed.

**Results:**

In contrast to previous reports for eukaryotic MBF1 (i.e. *Arabidopsis thaliana*, insect and human) the two archaeal MBF1 orthologs, TMBF1 from the hyperthermophile *Thermoproteus tenax *and MMBF1 from the mesophile *Methanosarcina mazei *were not functional for complementation of an *Saccharomyces cerevisiae *mutant lacking Mbf1 (*mbf1*Δ). Of twelve chimeric proteins representing different combinations of the N-terminal, core domain, and the C-terminal extension from yeast and aMBF1, only the chimeric MBF1 comprising the yeast N-terminal and core domain fused to the archaeal C-terminal part was able to restore full wild-type activity of MBF1.

However, as reported previously for *Bombyx mori*, the C-terminal part of yeast Mbf1 was shown to be not essential for function. In addition phylogenetic analyses revealed a common distribution of MBF1 in all Archaea with available genome sequence, except of two of the three Thaumarchaeota; *Cenarchaeum symbiosum *A and *Nitrosopumilus maritimus *SCM1.

**Conclusions:**

The absence of MBF1-interacting activators in the archaeal domain, the presence of a Zn ribbon motif in the divergent N-terminal domain of aMBF1 and the complementation experiments using archaeal- yeast chimeric proteins presented here suggests that archaeal MBF1 is not able to functionally interact with the transcription machinery and/or Gcn4 of *S. cerevisiae*. Based on modeling and structural prediction it is tempting to speculate that aMBF1 might act as a single regulator or non-essential transcription factor, which directly interacts with DNA via the positive charged linker or the basal transcription machinery via its Zn ribbon motif and the HTH domain. However, also alternative functions in ribosome biosynthesis and/or functionality have been discussed and therefore further experiments are required to unravel the function of MBF1 in Archaea.

**Reviewers:**

This article was reviewed by William Martin, Patrick Forterre, John van der Oost and Fabian Blombach (nominated by Eugene V Koonin (United States)). For the full reviews, please go to the Reviewer's Reports section.

## Background

Although the Archaea, the third domain of life, are prokaryotes, the processes involved in genetic information processing, including transcription, are more similar to their eukaryotic counterparts. The minimal archaeal transcription machinery consists of a multi-subunit RNA-polymerase (RNAP) resembling the eukaryotic RNA polymerase II, a TATA-box binding protein (TBP) that is also similar to its eukaryotic counterpart, and transcription factor B (TFB), which is homologous to eukaryotic (RNAP) TFIIB [1]. Multiple forms of the general transcription factors (GTFs), TBPs and TFBs, are commonly found in Archaea, and it has been proposed that they might function similar to bacterial sigma-factors, which regulate transcription in response to environmental changes [2]. However, relatively little is known about transcriptional regulation in Archaea and how it helps to confer fitness across a broad range of environments, including hostile ones.

Given the eukaryotic like- nature of the basal transcription machinery in Archaea, it is striking that most transcriptional regulators are bacterial-type regulators, modulating a eukaryotic-type core transcription apparatus. Only few eukaryotic-type transcriptional regulators were reported so far [3,4]. Multiprotein-bridging factor 1 (MBF1), known as transcriptional co-activator in Eukaryotes, has been identified in Archaea and therefore seems to be present in all organisms, which harbor TBP as GTF, Archaea and Eukaryotes [3], raising questions about its possible function in Archaea.

MBF1 was first purified from posterior silk gland extracts of the silkworm, *Bombyx mori *and was subsequently found in organisms as distant as mammals, *Arabidopsis *and yeast [5-24]. In insects *in vitro *transcription studies revealed that MBF1 activates transcription of the *fushi tarazu *(*ftz*) gene by bridging the activator FTZ-F1 (nuclear hormone receptor) and TBP in *B. mori *and *Drosophila melanogaster *[5-7]. The *ftz *gene of *D. melanogaster *is expressed during embryogenesis and metamorphosis and is connected with body segmentation [18]. In general, MBF1 mediates transcriptional activation by bridging between TBP and either steroid/nuclear hormone receptors (e.g. FTZ-F1 in insects [5-7], Ad4BP/SF-1 in human [8]) or leucine zipper (bZIP)-type transcriptional activators such as Gcn4 in yeast [9] and ATF1, c-Jun, and c-Fos in human [8,17]). Therefore MBF1-dependent activators are responsible for the regulation of numerous different processes in these organisms [5 - 24, for recent review see [25]]. A unique structural feature of MBF1 interacting activators is that they contain a conserved basic region in their DNA-binding domains. It has been previously suggested that the co-activator MBF1 mediates transcriptional activation by interaction with the conserved basic region, which stimulates the binding of the co-activator-activator complex to the target DNA sequences, in a similar manner as the human T-cell leukaemia virus transactivator Tax does [25-27].

In yeast, Mbf1 interacts with Gcn4 and Tbp directly and is involved in histidine synthesis, by mediating the Gcn4-dependent transcriptional activation of the *HIS3 *gene, which encodes imidazole-3-phosphate dehydratase [7]. Yeast mutants lacking either *MBF1 *or *GCN4 *are viable, but are defective in *HIS3 *activation, and they therefore exhibit sensitivity to 3-aminotriazole (3-AT), an inhibitor of the *HIS3 *gene product [7,28].

Apart from the well-documented role of eukaryotic MBF1 in transcription, it has been speculated that yMbf1 might be involved in translation fidelity [[29], see reference 25 for review]. It was found that yeast strains with a deletion of *MBF1 *(*mbf1*Δ) exhibit an increased rate of ribosomal +1 frameshifting for several different reporter gene constructs harboring frameshift mutations [29]. It has been suggested that the interaction between the truncated product of Mbf1 and Tbp is impaired, thus affecting the RNAP III dependent transcription of tRNA genes. The resulting reduced levels of tRNAs are supposed to induce increased rates of frameshift misreading, leading to suppression [29]. Alternatively, Koning and co-workers (2009) suggested that MBF1 might also be involved in the biogenesis of ribosomes or tRNA, possibly indirectly by transcription regulation of factors contributing to these pathways [25]. However, up to now, no experimental data support the connection of MBF1 with RNAP III transcription initiation or translation.

Previous studies revealed that expression of each of the three paralogues of MBF1 from the plant *Arabidopsis thaliana *was able to restore Mbf1 function in the yeast deletion strain (*mbf1*Δ)[21]. Moreover, the defect of *mbf1*Δ was also rescued by expression of silkworm or human MBF1 (Takemaru and Hirose, unpublished observation [7]) suggesting a common phylogenetic conserved function of MBF1 within Eukaryotes. On the basis of a combined bioinformatic and experimental approach, a co-evolution of MBF1 and TBP has been suggested and relevant residues for TBP and MBF1 interaction were predicted for Archaea, protists, plants, fungi and animals [30]. However, so far no experimental data are available for the biological function of MBF1 in Archaea.

In the present study, it was tested whether a*MBF1 *from *Thermoproteus tenax *(T*MBF1*) and *Methanosarcina mazei *(M*MBF1*) as well as constructed archaeal-yeast chimeric proteins were able to complement the function of yeast *MBF1 *as an attempt to elucidate the possible function of MBF1 in Archaea and to study the evolutionary conservation of MBF1 and of MBF1 domains.

## Results and Discussion

### Eukaryotic and archaeal MBF1: a comparison

Database searches (BLASTX) revealed sequences with apparent MBF1 similarity in almost all available 64 archaeal genomes (Status: 15.12.2010). Only in two of the three members of the recently proposed phylum Thaumarchaeota, *Cenarchaeum symbiosum *A and *Nitrosopumilus maritimus *SCM1, MBF1 is absent [25], whereas *Candidatus Nitrososphaera gargensis *possesses a single MBF1 homolog. All genomes harboring MBF1 contain a single MBF1 homolog with the only exception of Halobacteriales, which harbor two paralogues [25].

In general MBF1 comprises an N-terminal domain that is connected by a flexible linker to the C-terminal part, which is composed of a HTH domain and a short C-terminal stretch (Additional File 1). Whereas the HTH domain of MBF1 is well-conserved in Archaea and Eukaryotes and comprises four α-helices, the N-terminal domain as well as the C-terminus is more divergent [3,25]. The N-terminal domains of aMBF1 and eukaryotic MBF1 reveal no obvious similarity and thus are non-orthologous. In the N-terminal part all aMBF1 s possess a well-conserved Zn ribbon motif, predicted on the basis of two pairs of cysteine residues, which is absent in eukaryotic counterparts (Additional File 1). Due to the presence of the Zn ribbon motif a possible direct binding of aMBF1 to DNA has been suggested previously [3,25]. The predicted Zn ribbon motif in archaeal MBF1 is similar to the reported non-classical zinc finger-like domain, which is also present in other transcription-related proteins from Archaea and Eukaryotes, including *Pyrococcus furiosus *transcription factor B (*Pfu*TFB), human and yeast transcription factor IIS (TFIIS), *Thermococcus celer *RNAP II subunit M (*Tc*RPOM) and human TFIIB [31-35]. The presence of the non-classical zinc finger-like domain in aMBF1 therefore suggests a possible direct interaction with the RNAP rather than DNA binding. The HTH domain in the C-terminal part of MBF1 is highly conserved in Archaea (Additional File 1) and it has been predicted as the only highly conserved cro-HTH domain vertically inherited from Archaea to Eukaryotes [3]. The linker (residues 35-62 in *Bm*MBF1 and residues 41-68 in yMbf1) exhibits less prominent conservation and in *B. mori *a functional role rather than an architectural was predicted due to its flexible structure [7]. In Archaea this essential region encompasses a high level of basic residues (arginine or lysine) (Additional File 1). For the C-terminal stretch, as shown in Additional File 1, all aMBF1, except Thermoplasmatales, the Thaumarchaeon *C. N. gargensis *and halobacterial MBF1b paralogues (NP2072A, VNG1483C, rrnAC0872, HQ2874A), comprise a C-terminal extension with a well-conserved four amino acid motif "[TS]-[LIVMF]-G-[DENI]".

The regions of MBF1 required for protein-protein interaction with TBP and the sequence specific activator have been identified in insects and in yeast [7,9]. Deletion analyses of *B. mori *MBF1 and yMbf1 demonstrated that the core domain of MBF1 comprising the flexible linker and HTH domain (residues 35-113 and residues 41-119, respectively) is essential for the binding of TBP and the transcriptional activators, FTZ-F1 in *B. mori *[7] and Gcn4 in yeast [9]. Further analysis revealed that mainly the conserved HTH domain in *Bm*MBF1 (residues 65-132) and yMbf1 (residues 71-138) contribute to these interactions. A deletion of the N-terminal part (residues 1-66) as well as of the C-terminal part (residue 114-146) in *Bm*MBF1 still retains significant activity. NMR studies (hMBF1, *Bm*MBF1 [36,37]) revealed the presence of four amphipathic helices in the highly conserved HTH region (residues 63-132) in *Bm*MBF1, that are bundled into a compact form [36] and a function in maintaining domain stability has been proposed. Binding of TBP in hMBF1 occurs via the amphipathic helices in the HTH domain [37] and in yeast an aspartate residue at position 112 (Asp 112), located in helix three, was identified as important binding site for Tbp [9]. The N-terminal domain of MBF1 from *Bm*MBF1, yMbf1 and hMBF1 is not required for binding TBP. In insects and yeast, binding of TBP to a truncated version of MBF1 lacking its N-terminal amino acids (residues 1-34 in *Bm*MBF1 [7] and residues 1-40 in yMbf1 [9]) remained unaffected but the binding of the activator, Gcn4 or FTZ-F1, was slightly reduced. Therefore both, the N-terminal region and the core domain are indispensable for binding the activator, whereas the HTH domain is essential for binding TBP. The observation that the N-terminal domain of MBF1 is not required for binding TBP in human, yeast and insects [6,7,9] suggests that at least aMBF1 and yTbp interaction is possible, despite the divergent N-terminal region with a conserved Zn ribbon motif.

**Figure 1:**
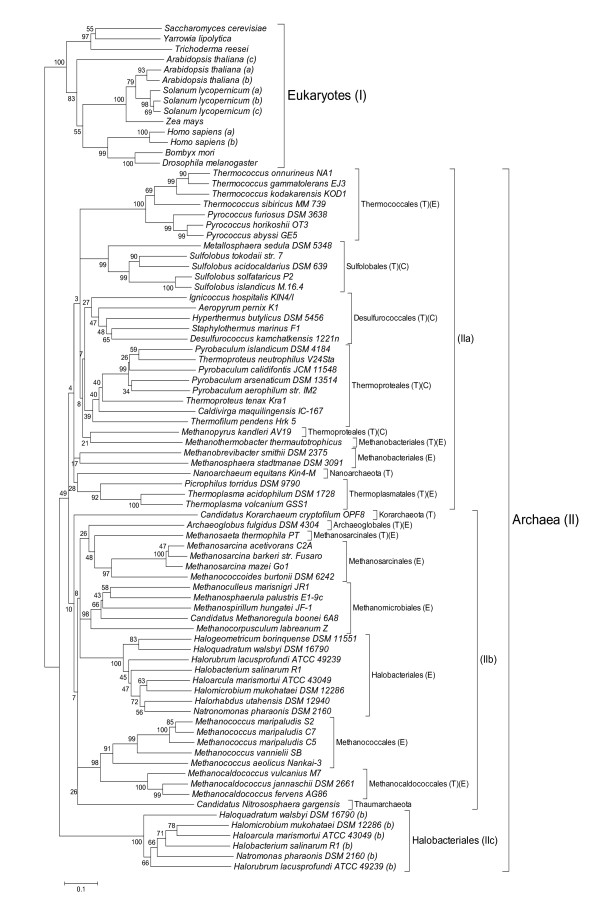
**Phylogenic tree of archaeal and eukaryotic MBF1s**. The phylogenetic tree was constructed based on the multiple alignment of the MBF1 sequences lacking the non-orthologous N-terminal domain of Archaea and Eukaryotes (residues 1-37 of *Ttx*MBF1, Additional file 1). The tree was constructed based on pairwise distance estimates of the expected number of amino acid replacements per site using MEGA4 software [69]. Bar = 0.20 amino acid replacement per site. The bootstrap values are indicated to the left of the branches. Branch length indicates the relative evolutionary distances. T: Thermophile, C: Crenarchaeota, E: Euryarchaeota.

A phylogenetic tree (distance-based neighbour-joining) was constructed based on the multiple alignment of the MBF1 sequences lacking the non-orthologous N-terminal domain of Eukaryotes and Archaea (residues 1-37 of *T. tenax *MBF1) (Additional File 1). Bootstrap data supported two distinct branches: (I) MBF1 homologues of Eukaryotes and (II) MBF1 homologues of all Archaea (Figure 1). The archaeal branch II is subdivided into three clusters, the first one (IIa) includes MBF1 homologues from Crenarchaeota, Nanoarchaeota and part of the Euryarchaeota (i.e. Thermococcales, Thermoplasmatales and Methanobacteriales). The second archaeal branch (IIb) comprises MBF1 homologues of the Korarchaeon *C. K. crytophilum*, the Thaumarchaeon *C. N. gargensis *and all the residual Euryarchaeota. The third archaeal branch (IIc) harbours the halobacterial MBF1b paralogues. Within the Halobacteriales there are two distinct lineages of MBF1, indicating that gene duplication occurred at an early stage of halobacterial evolution and that the MBF1 paralog (branch IIc) might have acquired new functions.

Noteworthy most of the archaeal (hyper)thermophiles, are grouped in branch IIa and only few (hyper)thermophiles (*Methanosaeta thermophila*, *Archaeaglobus fulgidus*, *C. K. crytophilum *and the three Methanocaldococcales) are found in branch IIb (Figure 1). Therefore, it is tempting to speculate that adaptation of MBF1 to high temperature might have been an important selection factor in branch IIa. Although no general rule could be proposed for protein stabilization at high temperature, several recurrent themes and general tendencies were observed in a large-scale comparative analysis of proteins from closely related mesophilic and thermophilic *Methanococcus *species [[38], for review [39]]. In addition it has recently been suggested that protein misfolding plays a key role in determining evolutionary rates [40-44]. Many proteins require the assistance of molecular chaperones for correct folding and it has been proposed that -GroEL-dependent folding increases evolutionary rates by buffering the harmful effects of misfolding-related mutations [44]. The protein folding apparatus of Archaea contains both eukaryotic and bacterial components [45], however, so far no information of the evolutionary effects of chaperones on protein evolution in Archaea is available.

As reported previously the genomic context of *mbf1 *is well conserved in Crenarchaeota and comprises genes, which encode proteins with predicted function in informational processing such as transcription or translation. The *mbf1 *gene is in close neighborhood to the genes *pan *(proteasome-activating nucleotidase), *hflX *(G-protein of the HflX family) [46], *tgt *(tRNA guanine transglycosylase), *tfb *and *tfe *(coding for archaeal homologues of transcription factor II B and the N-terminal half of alpha - subunit of transcription factor II E, respectively) and *rpoG *(hypothetical RNA-polymerase subunit G) [25]. In Euryarchaeota, the genomic context of *mbf1 *is less conserved; however, proximity to *pan *is also observed. In Thaumarcheaota, *N. maritimus *SCM1 and *C. symbiosum *A, *mbf1 *is replaced by a gene encoding a putative phosphate-uptake regulator and the genomic context in the close neighborhood is similar to Crenarchaeota [25]. Unfortunately, so far the complete genome of *C. N. gargensis *is not available for comparison.

### **Complementation of yeast mbf1 **Δ **by archaeal MBF1**

Transcription regulation in Archaea is not well understood. Although the archaeal transcription apparatus is similar to the eukaryotic one; most archaeal transcription regulators are of the bacterial-type [4,47,48]. MBF1 is highly conserved in Eukaryotes and Archaea and a co-evolution with TBP has been proposed previously [24]. However, the function of co-activators from Archaea has not been studied so far and the biological role of aMBF1 as multiprotein bridging factor has not been documented. Therefore a key question concerning aMBF1 is its *in vivo *function. To address this issue, complementation studies in yeast were performed and it was tested whether aMBF1 s from the hyperthermophile *Thermoproteus tenax *and the mesophile *Methanosarcina mazei *are functional for complementation in yeast lacking Mbf1 (*mbf1*Δ). The mesophilic *M. mazei *MBF1 was included in this study in order to exclude temperature-dependent effects, which might be caused by expression in a mesophilic host (e.g. incorrect folding, missing activity).

In yeast, Mbf1 is involved in the co-activation of the transcription of the *HIS3 *gene, encoding the third enzyme of histidine biosynthesis, by bridging between Tbp and the bZIP type activator, Gcn4 [9]. Deletions either of *MBF1 *or *GCN4 *in yeast are viable, but sensitive to 3-AT. Therefore complementation by eukaryotic MBF1 could be easily monitored by growth in the presence of 3-AT, as demonstrated previously for the three *A. thaliana *MBF1 s [21].

**Figure 2:**
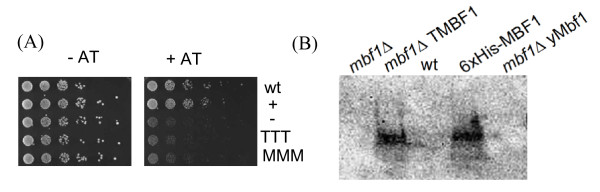
**Functional complementation of yeast *mbf1*Δ by archaeal MBF1 from *T. tenax *(T) and *M. mazei *(M)**. (A) AT sensitivity of yeast wild type (WT), the *mbf1*Δ strain carrying py*MBF1 *(+), empty vector (-), pT*MBF1 *(TTT) and pM*MBF1 *(MMM) using the vector pRS316 under control of the natural yeast MBF1 regulatory region. Serial dilutions of the respective strains were placed on minimal medium either in the presence (+AT) or absence (-AT) of 3 mM aminotriazole. The plates were incubated at 30°C for 3 days. (B) Expression of TMBF1 protein (pHRT*MBF1*) in the y*mbf1*Δ strain was detected by immuno blotting using a polyclonal antibody against TMBF1.

First experiments were performed with the expression vector, pHR98/3, under the control of a constitutive and high-level expression promoter. The expression plasmids encoding full-length TMBF1 and MMBF1 were introduced into the *mbf1*Δ strain and the AT sensitivity was compared to that of the wild-type and the *mbf1*Δ strain expressing either yeast Mbf1 (pHRy*MBF1*, positive control) or an empty vector (pHR98/3, negative control) (data not shown). As expected, growth of the *mbf1*Δ strain was sensitive to 3-AT, and 3-AT resistance was restored by re-introducing yeast Mbf1 on a plasmid (pHRy*MBF1*, positive control). However, the growth of the *mbf1*Δ strain in presence of 3-AT was not restored by introducing the expression plasmid comprising full-length aMBF1 from *M. mazei *or *T. tenax*, respectively. In order to exclude deleterious effects caused by overexpression of aMBF1 in yeast, the experiments were repeated using the vector pRS316. The three genes, *yMBF1*, *TMBF1 *and *MMBF1*, were introduced under the control of the endogenous yeast *MBF1 *regulatory region (py*MBF1*, pT*MBF1 *and pM*MBF1*, respectively). However, as shown in Figure 2A also under control of the 'natural' *MBF1 *promoter no complementation by aMBF1 as well as by the empty vector (pRS316, negative control) was observed, whereas yeast Mbf1 restored 3-AT sensitivity of the *mbf1*Δ strain.

Western blot analysis of yeast cell extracts, using polyclonal antibodies generated against TMBF1, confirmed that TMBF1 (pHRT*MBF1*) was successfully expressed in the *mbf1*Δ strain (Figure 2B). Thus, the lack of complementation appears to be not due to the absence of MBF1 expression using the high level expression vector. For the vector pT*MBF1*, TMBF1 expression was only hardly detectable with the antibody (data not shown). The antibody showed no cross-reactivity with recombinant MMBF1 or yeast Mbf1. The addition of tags (i.e. positively charged His-tag) for protein detection was consciously avoided, since they might interfere with protein-protein interaction of MBF1 with Tbp and Gcn4, required for functional complementation in yeast.

In general, many different recombinant archaeal proteins of (hyper)thermophilic origin have been successfully and functional expressed in mesophilic hosts, such as, *Escherichia coli*, *Bacillus subtilis *and yeast [49]. More recently TFB1, TFB3 and all the subunits of RNAP from *Sulfolobus solfataricus *P2 were efficiently expressed in yeast and used for yeast-two-hybrid experiments [50,51]. Furthermore, of great interest for this study, i) the RNAP subunit P from the hyperthermophilic Euryarchaeon *Pyrococcus furiosus *functionally replaced the eukaryotic polymerase subunit Rbp12 in the respective yeast mutant [52] and ii) yeast, human, and archaeal TBP are functionally interchangeable *in vitro *in a *Methanococcus*-derived cell-free transcription system [53].

Therefore, these results indicate that aMBF1, in contrast to eukaryotic MBF1 from human, insects [7] or plants [21], is not able to complement and to restore eukaryotic MBF1 wild-type function (3-AT resistance) in yeast by substituting for eukaryotic MBF1 during Gcn4-mediated transcription activation.

Based on a bioinformatic and mutational approach in yeast Liu and co-workers [30] suggested the co-evolution of Mbf1 with Tbp in Eukaryotes and Archaea. Aspartic acid (position 112) in yMbf1 and glutamine (position 68) in yTbp were identified as important residues required for Mbf1:Tbp interaction during Gcn4-dependent transcriptional activation [9,30]. In aMBF1, they identified lysine, arginine, serine, or asparagine as interacting residues that correspond to yMbf1 D112 based on the multiple alignment of 21 full-length Mbf1 protein sequences [30]. An *in vivo *mutational approach for yMbf1 revealed that combinations that occur naturally (e.g. the mutant yMbf1-D112R (WT-Tbp-68Q); Archaea) showed no effect, whereas combinations not normally found, like yMbf1-D112K (WT-Tbp-68Q), were sensitive to 3-AT, indicating that the interactions between yMbf1 and yTbp were disrupted in the latter mutants [30]. The amino acids corresponding to position 112 in TMBF1 and MMBF1, are arginine (position 102) and lysine (position 108), respectively (Additional File 1), suggesting that TMBF1 but not MMBF1 might be able to interact with yTbp. However, as shown above TMBF1 was not able to complement the yeast *mbf1*Δ strain in spite of the presence of the appropriate amino acid for the interaction with yTbp.

In order to further analyze MBF1 and TBP interaction sites in Archaea a multiple sequence alignment of 14 eukaryotic and all available 68 archaeal MBF1 and TBP sequences was performed (CLUSTAL_X, [54]) focusing on the determined interaction sites of yeast (Mbf1-D112, Tbp-Q68) [30]. A summary of compensatory amino acid changes in the MBF1:TBP interaction sites is shown in Additional File 2 and 3. Based on more archaeal genomes available the comparative analysis suggests that other additional TBP:MBF1 interacting residues than previously reported [30] are present in Archaea and thus the diversity of archaeal residues is much higher than previously predicted. However, the proposed co-evolution of MBF1:TBP, the importance of the well-conserved HTH-domain of MBF1 for TBP interaction and the fact that archaeal TBP and eukaryotic Tbp are functionally interchangeable (*in vitro *transcription system [53]) suggests that the interaction between MBF1 and TBP might be also conserved in Archaea.

Beside aMBF1-yTbp interaction also the binding of aMBF1 to the activator Gcn4, which depends on both the N- terminal and HTH- domain is important. In yeast the binding of Gcn4 to Mbf1 was not altered by a D112A mutation (Tbp binding), but it was slightly reduced by the N-terminal deletion of yMbf1 (*MBF1*ΔNT). Both mutants (D112A and *MBF1*ΔNT) are sensitive to 3-AT as shown by Takemaru and co-workers [9]. It has been reported previously that Archaea lack bZIP-proteins like the transcriptional activator Gcn4 [25]. In accordance with previous analysis PSI-BLAST searches with yGcn4 (281 amino acids) and *Bm*FTZ-F1 (534 amino acids) in all available archaeal genomes revealed no homologues to the activators which interact with MBF1. Therefore, it is questionable if the respective sites for Gcn4 interactions are present in aMBF1 either in the divergent N-terminal domain or in the conserved HTH domain. In summary, these data suggest that the interaction of aMBF1 with yGcn4 could be impaired rather than with yTbp and in the light of the above, chimeric proteins of Archaea and yeast were constructed and tested for 3-AT sensitivity.

### Chimeric yeast - archaeal MBF1 variants

Studying protein function by interchanging protein domains is a powerful approach that exploits the natural variability of protein structure. Mbf1 from yeast exhibits high structural similarity with *T. tenax *and *M. mazei *MBF1, despite their difference in amino acid sequence; TMBF1 and MMBF1 share 39% and 36% amino acid identity with their yeast counterpart, respectively.

**Figure 3:**
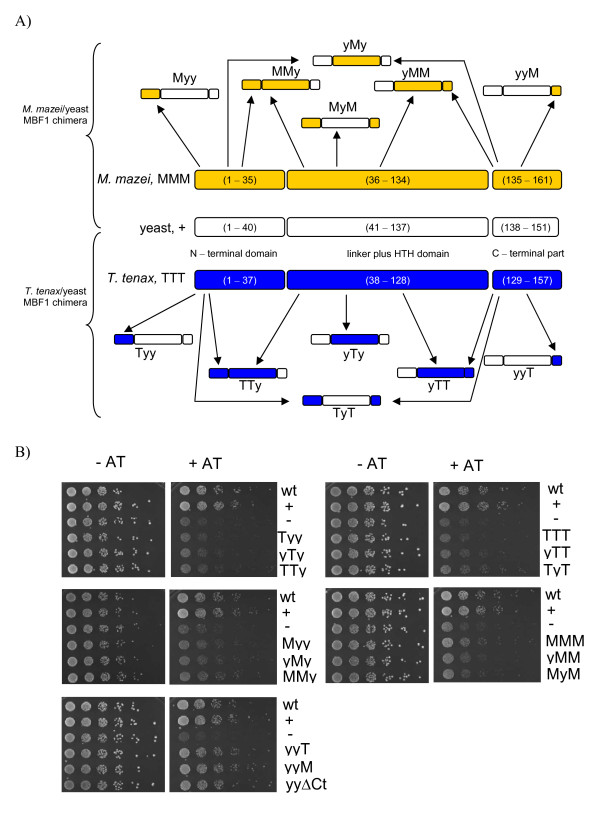
**Schematic overview of the MBF1 from yeast, *T. tenax *and *M. mazei*, and constructed chimeric Archaea/yeast MBF1 proteins**. (A) Chimeric proteins were generated by the use of recombination/gap repair cloning technique and the vector pRS316 under control of the natural yeast MBF1 regulatory region. (B) Functional complementation of yeast deletion mutant by yeast Mbf1 (y), aMBF1 (*T. tenax *(T) and *M. mazei *(M)), and chimeric MBF1 variants. Only *mbf1*Δ expressing MBF1 comprising the C-terminal extension of *T. tenax *(yyT) or *M. mazei *(yyM) MBF1 restored WT activity, i.e. AT resistance. The yyΔCt yMBF1 mutant lacking the C-terminal part (residues 138-151) was used as control. The assay was performed as described above (see Figure 2).

The observed missing complementation of aMBF1 in yeast might be caused by the presence of the Zn ribbon motif in the N-terminus of aMBF1 or as discussed above by changes of important amino acid necessary for Tbp and Gcn4 interaction. To test this possibility and to study the evolutionary conservation of MBF1 domains in Eukaryotes and Archaea, in total 12 chimeric interspecies MBF1 variants were constructed. Each chimeric protein was composed of different combinations of the N-terminal domain, the core domain (including the flexible linker and HTH-domain) and the C-terminal extension from either the hyperthermophile *T. tenax *or the mesophile *M. mazei*, and yeast (Figure 3A). The hybrid proteins were constructed using the recombination/gap repair cloning technique in yeast and 3-AT sensitivity in the *mbf1*Δ background was examined (Figure 3B). No complementation was observed for any of the aforementioned chimeric MBF1 variants targeting the N-terminal domain or the core domain (i.e. Tyy and Myy; yTy and yMy; TTy and MMy, yTT and yMM; TyT and MyM), indicating that neither the archaeal N-terminal nor the core domain, harboring the conserved HTH domain, was sufficient to allow for functional Mbf1 in yeast.

Next it was studied whether the well conserved, archaeal C-terminal extension has an influence on yMbf1 function. The *mbf1*Δ expressing the chimeric proteins, yyT- and yyM-MBF1, showed similar 3-AT resistance as the wild-type and the positive control (py*MBF1*, Figure 3B). However, also the *yMBF1 *mutant (yyΔCt -MBF1 (residues 1 - 137), control) lacking the yeast C-terminal part (residues 138 - 151) restored WT activity, i.e. 3-AT resistance (Figure 3B). This observation clearly indicates that the archaeal C-terminal stretch comprising the well conserved archaeal motif does not interfere with domain stability of yMbf1 (Figure 3B) and moreover that the C-terminal part of yeast MBF1 is not required for function as shown previously for the *Bm*MBF1 mutant Δ114-146 [7]. The TMBF1 antibody showed no cross-reactivity with the hybrid proteins, however, the functional complementation by yyT and yyM nicely demonstrates functional expression.

In summary, complementation studies with chimeric archaeal/yeast proteins indicate that neither the N-terminal, nor the core domain of archaeal MBF1 is able to substitute for the respective eukaryotic domains. Only the archaeal and yeast C-terminal extension, which was shown here to be dispensable for yMbf1 function, can be successfully swapped. Therefore, due to the fact that the N-terminal and core domain of yeast are required for Gcn4 interaction [9], MBF1 interacting activators are absent in archaeal genomes and conserved MBF1:TBP interaction has been suggested [30], it is tempting to speculate that archaeal MBF1 is not able to bind to the yeast activator Gcn4 rather than yTbp.

### Computational analysis

#### Structure prediction of MBF1

**Figure 4:**
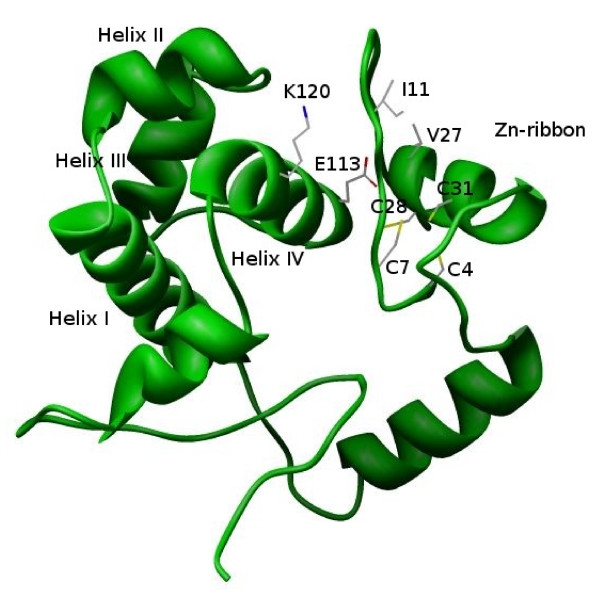
**Three dimensional model of *T. tenax *MBF1**. The figure was generated using the CHIMERA program [71]. The residues predicted to contribute to domain stability by van der Waals interactions are I11:K120, V27:E113 and the Zn ribbon motif (C4, C7, C28, C31) are indicated.

Since no 3 D structure of MBF1 is available, the previously described solution structure of *T. reesei *C-terminal HTH domain [55] was used for modeling of *T. tenax *MBF1 via METASERVER, a consensus fold-recognition method [56], PHYRE [57], I-TASSER [58] fold-recognition and *ab initio *methods. These three methods suggested different HTH domains as the best template for *T. tenax *MBF1 with both, transcriptional regulator from *Vibrio cholerae *(PDB ID: 1y9q) and C.BclI a control element of the BclI restriction-modification system in *Bacillus caldolyticus *(PDB ID: 2b5a) as best hits, which score significantly above the threshold. The human MBF1 (endothelial differentiation-related factor 1 protein, EDF-1; PDB ID: 1x57) and the regulator of cytolysin operon in *Enterococcus faecalis *(PDB ID: 1utx) were identified simultaneously by at least two methods. Only I-TASSER [58] provided a 3 D model for the complete MBF1 sequence. The structural models were analyzed by different structure validation programs including PROCHECK [59] for the evaluation of the Ramachandran plot quality, WHATCHECK [60] for the calculation of packing quality Z-score, and VERIFY-3 D [61] for the evaluation of sequence-structure compatibility (Table 1). In general, quality values obtained for the 3 D models are similar to those observed in the template structures indicating a high quality of the MBF1 low-resolution model presented in this work (Figure 4).

**Table 1:** Evaluation of the constructed three-dimensional model of archaeal MBF1 from *Thermoproteus tenax *(TMBF1)

Criteria	Characteristic	MBF1 (M1-E157)	MBF1 (R64-N141)	2b5a (A)	1y9q (A)	1x57 (A)	1utx (A)
PROCHECK	Most favored regions	60.0%	94.1%	97.1%	93.1%	84.7%	93.3%
	
	Additional allowed	24.4%	5.9%	2.9%	6.3%	11.1%	6.7%
	
	Generally Allowed	8.9%	0%	0.0%	0.6%	2.8%	0.0%
	
	Disallowed regions	6.7%	0%	0.0%	0.0%	1.4%	0.0%

WHATCHECK (Z-scores)	2nd generation packing quality	-4.061	-0.752	-0.1	-0.9	-1.758	-0.7

VERIFY-3D	3D-1 D score (≥0.2)	72.15%	64.56%	84.62%	98.28%	96.74%	100%

PDB ID: 2b5a, C.BCLI, control element of the BCLI restriction modification system (*Bacillus caldolyticus*); PDB ID: 1y9q, HTH_3 family transcriptional regulator (*Vibrio cholerae*); PBD ID: 1x57, EDF-1, human multiprotein bridging factor 1 α; PDB ID: 1utx, CYLR2, Transcriptional repressor of cytolysin operon (*Enterococcus faecalis*).

Analysis of the three-dimensional model of TMBF1 suggests two residues in the N-terminal Zn ribbon motif that establish van der Waals interactions with residues in the HTH domain: (i) The isoleucine residue at position 11 (Zn ribbon) interacts with lysine at position 120 (helix-IV) (Figure 4). TMBF1-I11 is highly conserved in archaeal sequences and TMBF1-K120 is well conserved in both, Archaea and Eukaryotes (see Additional File 1). In yeast Mbf1, lacking the Zn ribbon motif, TMBF1-I11 is substituted by alanine. (ii) The valine at position 27 (Zn ribbon motif) which is well conserved in Archaea interacts with the not so well conserved E113 (helix-IV) of TMBF1 (Figure 4). These predicted interactions between the Zn ribbon motif and the HTH domain would be favorable for the folding stability of *T. tenax *MBF1.

#### Prediction of functional residues of archaeal MBF1

**Figure 5:**
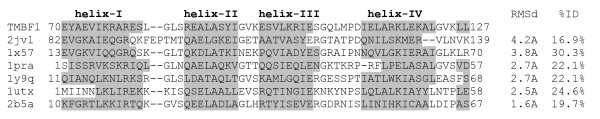
**Structural superimposition of the conserved helix-turn-helix (HTH) domains**. The structural superimposition was performed using the CE method [72]. Alpha helical structural elements are highlighted by grey shading according to DSSP [73]. The PDB IDs are: 2jvl, C-terminal domain of MBF1 (*Trichoderma reesei*); PBD ID: 1x57, EDF-1 (human Multiprotein bridging factor 1 α); PDB ID: 1pra, DNA-biding domain (residues 1 to 69) of the bacteriophage 434 repressor; PDB ID: 1y9q, HTH_3 family transcriptional regulator (*Vibrio cholerae*); PDB ID: 1utx, CYLR2, Transcriptional repressor of cytolysin operon (*Enterococcus faecalis*); PDB ID: 2b5a, C.BCLI, control element of the BCLI restriction modification system (*Bacillus caldolyticus*).

Comparison of the HTH domain from the 3 D model of TMBF1 with the C-terminal domain of *Trichoderma reesei *MBF1 (PDB ID: 2jvl, [55]) revealed that helix-II is the most highly conserved region (Figure 5). The C-terminal domain of *T. reesei *MBF1 [55] has been reported previously to be structurally similar to the DNA-binding domain (residues 1 to 69) of the phage 434 repressor (Φ434rep) (PDB ID: 1pra). For Φ434rep the residues involved in DNA binding were identified; Φ434rep-Q17 in helix-II participates in the anchoring to the DNA and Φ434rep-R43 in helix-IV inserts in the DNA minor groove [62]. In TMBF1, TMBF1-R86 at the first position of helix-II corresponds to Φ434rep-Q17 and TMBF1-E113 in helix-IV to Φ434rep-R43. These characteristics would be unfavorable for DNA binding, and concurs with previous studies that *Bm*MBF1 does not bind directly to DNA, but instead stabilizes FTZ-F1-DNA interactions [6]. In *Bm*MBF1 Q90 and I117 are at the respective positions (Additional File 1). DNA phosphate interaction with positive charged residues has been previously described for Φ434rep [62]; arginine and lysine residues positioned in the loop II connecting helices III and IV of Φ434rep establish important contacts with DNA phosphates [62]. However, the respective positive charged residues are absent in the HTH domain of *B. mori*, *T. reesei *and *T. tenax *MBF1 suggesting no binding to DNA via the core domain. Therefore, the HTH domain in archaeal MBF1 might be important for protein-protein interaction, possibly with TBP which is well conserved also in Archaea.

It has been suggested previously [7] that the flexible linker in eukaryotic MBF1 could have a functional role rather than an architectural. In all aMBF1 a well- conserved lysine-arginine-rich region is present between the Zn ribbon motif and the HTH domain, which would be favorable for the interaction with the DNA phosphate backbone (Additional File 1). In eukaryotic MBF1 lacking the Zn ribbon motif, this lysine-arginine-rich region is absent (Additional File 1). Taken together, based on predictions derived from modeling as well as structural analyses, and the performed complementation studies, it is tempting to speculate that aMBF1 might act as a single regulator or non-essential transcription factor, which directly interacts with DNA via the positive charged linker or to the basal transcription machinery via its Zn ribbon motif (i.e. RNAP) and the HTH domain (i.e. TBP).

#### Origin of archaeal and eukaryotic MBF1: trends in evolution

As previously indicated [3], there is an extensively diversity of HTH domains in Archaea as well as in Bacteria. Most of the predicted archaeal HTH-containing proteins show significantly higher similarity to bacterial than to eukaryotic HTH-containing transcription factors, with the only exception of the core transcription machinery comprising eukaryotic-like transcription factors such as, TFIIE-α, TFIIB/TFB and MBF1 [3]. It has been proposed that beyond the core machinery, the bacterial-like HTH- containing archaeal proteins might have been established and maintained through multiple horizontal gene transfer events from Bacteria to Archaea in the course of evolution. Moreover, most of the archaeal HTH domains appear to have undergone at least some evolution within the archaeal superkingdom [3]. MBF1 is the only highly conserved HTH-domain in Archaea and Eukaryotes. Eukaryotic MBF1 is not a general transcription factor since the disruptants are viable and able to grow under different growth conditions [9]. The situation in Archaea appears to be similar, because MBF1 is well distributed but lost in two of the three members of the Thaumarchaeota, *C. symbiosum *A and *N. maritimus *SCM1. The absence of MBF1 homologs in Bacteria suggests that MBF1 was originally present in the last common ancestor of Archaea and Eukaryotes.

Eukaryotic MBF1 lacks the Zn ribbon motif and MBF1-dependent gene regulation requires the function of specific activators belonging to bZIP-like protein or steroid/nuclear-hormone receptor family, which bind directly DNA. In contrast, archaeal MBF1 exhibits a Zn ribbon motif and homologs of eukaryotic MBF1-specific activators are absent in Archaea, suggesting a different mode of action of MBF1. Further on, the conserved genomic context of *mbf1 *in the archaeal domain with proximity to genes involved in information processing proposes a basal function of aMBF1 in transcription or translation [25].

Two evolutionary scenarios can be discussed which might have evoked the present-day image. One possibility is that the common ancestor possessed an ancient Zn ribbon containing MBF1 version which interacts directly with DNA and/or RNAP and no specific interacting activators were required. This MBF1 version was maintained in Archaea but further evolved in Eukaryotes. Probably the Zn ribbon motif was lost due to the emergence of specific MBF1-interacting activators (bZIP like proteins or FTZ-F1 related proteins), which allowed for improved fine tuning of gene regulation in a more complex cellular network; for instance, gen *HIS3*, which possesses a constitutive "TATA-less control element" as well as a regulatory region ("TATA-containing control element") and the latter is regulated by the Gcn4-Mbf1 complex in yeast under starvation conditions [63]. An alternative possibility is that the last common ancestor harbored both an eukaryotic-like MBF1 and homologs of MBF1-interacting regulators. The specific interacting activators were lost in the archaeal lineage and provoked the evolution of the Zn ribbon containing archaeal specific MBF1, which acquired new functions.

The fact that MBF1 is involved in many different fundamental processes in Eukaryotes (e.g. histidine synthesis, development, immune and antioxidant defense, steroid/hormone synthesis, lipid metabolism) and interacts with different activators in a very sophisticated and complex manner seems to favor the first scenario. However, since the function of archaeal MBF1 is still unclear future studies have to be awaited, which will provide key insights into the evolution and mechanism of basic processes in both domains, Archaea and Eukaryotes.

## Conclusions

Previous studies with eukaryotic MBF1 revealed that the defect of a yeast *MBF1 *deletion was rescued by the expression of silkworm and human MBF1 [Takemaru and Hirose, unpublished observation, 7] or plant MBF1 (three *A. thaliana *paralogues [21]) indicating that MBF1 s from divergent eukaryotic sources are able to substitute yMbf1 in Gcn4-dependent transcriptional activation. In contrast the present study revealed that archaeal MBF1, neither from hyperthermophilic (Crenarchaeota) nor from mesophilic (Euryarchaeota) origin, was capable of functionally complementing the *mbf1*Δ in yeast. Furthermore, domain swap experiments suggest that the divergent archaeal N-terminal domain and the core domain of MBF1, harboring the conserved HTH domain, are not functional in yeast. This was surprising since the HTH domain of MBF1 is a cro-HTH type domain and has been identified previously as the only highly conserved, classical HTH domain that is vertically inherited in all Archaea and Eukaryotes. Further results obtained in this study indicated that the archaeal C-terminal extension containing the well conserved archaeal motif "[TS]-[LIVMF]-G-[DEN]" did not interfere with yMbf1 function and revealed that the C-terminal extension of yeast Mbf1 is not required for Mbf1 function (yyΔCt-MBF1).

The conservation of MBF1 among all organisms in which TBP is used as a general transcription factor suggests that MBF1 has a fundamentally important function also in Archaea. However, the absence of bZIP-like proteins in the archaeal domain, the presence of a non-classical type Zn ribbon motif, the positive- charged linker in aMBF1 and the complementation experiments using archaeal- yeast chimeric protein presented here suggest a different mode of action of MBF1 -may be as non-essential transcription factor or transcriptional regulator, which contacts DNA and/or the basal transcription apparatus probably via RNAP and/or TBP. Alternatively a role of MBF1 in translation either directly in ribosome biogenesis [25] or indirectly in translation fidelity [29] has been proposed. Thus, further studies (e.g. protein-protein interaction studies, mutational approaches) are required in order to elucidate the specific function of MBF1 in Archaea.

## Methods

### Yeast strains

Growth and manipulation of yeast was performed according to standard procedures. AEY3087 (*MAT***a ***his3*Δ*1 leu2*Δ0, *met15*Δ0, *ura3*Δ0) and an *mbf1*Δ*::kanMX *derivative thereof (Yeast Deletion Collection, ResGen/Invitrogen) were used to determine AT sensitivity. For this purpose, the strains were rendered His3^+ ^by transformation to histidine prototrophy with a *HIS3 *fragment.

### Cloning of MBF1 gene from yeast, *T. tenax *and *M. mazei*

To construct the overexpression vectors, pHRy*MBF1 *(YOR298C-A), pHRT*MBF1 *(TTX_1938, FR823290) and pHRM*MBF1 *(MM_1005) encompassing the coding region of yMbf1 and aMBF1 genes, the three DNA fragments corresponding to *yMBF1*, *TMBF1 *and *MMBF1 *open reading frame were amplified by PCR and then, the resulting DNA fragments were cloned into *Bam*HI and *Sal*I sites of pHR98/3 (2 μm, *URA3*) vector containing a constitutive and high-level expression promoter. The pairs of primers and the restriction enzymes used for cloning are indicated in Additional File 4.

To construct py*MBF1*, the 1,667-Kb *Eco*RI/*Not*I genomic fragment encompassing the entire *MBF1 *regulatory and coding region was cloned into pRS316 (2 μm, *URA3*) vector using the primer set yMBF1-yprom-r and yMBF1-yprom-f (Additional File 4). Plasmids encompassing the *MBF1 *gene from *T. tenax *(pT*MBF1*) and *M. mazei *(pM*MBF1*) were generated by recombination/gap repair cloning technique in yeast using py*MBF1 *as the starting vector (Addtional File 4). In these constructs, the yeast *MBF1 *open reading frame was replaced by the *MBF1 *gene from *T. tenax *and *M. mazei *to construct plasmids pT*MBF1 *and pM*MBF1*, respectively. Therefore, pT*MBF1 *and pM*MBF1 *encompass the yeast *S. cerevisiae MBF1 *promoter and the *MBF1 *coding region from *T. tenax *or *M. mazei*, respectively. After gap repair, plasmids were isolated from yeast by amplification in *E. coli *and verified by sequence analysis.

### Generation of chimeric MBF1s

Chimeric variants of yeast-archaeal MBF1 were constructed by gap repair in yeast as described above. They were named using a three letters code with y, T or M, corresponding to the origin from yeast (y), *T. tenax *(T) or *M. mazei *(M) and the order indicates the position, i.e. N- terminal domain, central region (flexible linker and helix-turn-helix- domain) or C- terminal extension (e.g. yTT). The chimeric variants were generated using PCR sewing (Additional File 5). For this purpose, two primer sets (external and internal) were designed to amplify the respective fragment of *MBF1 *with addition of sequences for gap repair. The external primers were designed encompassing the 50-bp target fragment in both outer arms. The gene- specific parts of the internal primers were designed to ensure that their 5'-termini matched 23-bp of the target sequence (Additional File 4). Gap repair and construct verification was performed as above.

### Generation of antibodies

Polyclonal antibodies against *T. tenax *MBF1 (anti-TMBF1) were generated against the His-tagged version of *T. tenax *MBF1 protein expressed in *Escherichia coli *(EUROGENTEC, Seraing, Belgium). SDS-PAGE and immuno blotting procedures were performed according to standard protocols. The TMBF1 antibody was used in a 1:500 dilution.

### Complementation test: Sensitivity to Aminotriazole

Yeast colonies of the wild-type (WT) and the *mbf1*Δ yeast strain transformed with plasmid expressing aMBF1, chimeric protein variants, yMBF1 or empty vector were grown in the appropriate minimal medium. For the serial dilutions, a suspension with an optical density (OD_600 _nm) of 0.3 was generated, and 5-fold serial dilutions thereof were made in a microtiter dish. The cells were transferred onto supplemented minimal medium with or without 3 mM aminotriazole using a 6 × 8 pronged replica plater. The plates were incubated for 3 days at 30°C.

### Computational analysis

All databases and software used in the present study are publicly available. Sequences of MBF1 and TBP from Archaea and Eukaryotes were retrieved from publicly available databases including Swiss-Prot (sp), GenBank (gb), EMBL (emb), and DDBJ (dbj). A list of Archaea with available complete genome sequence was retrieved from the Genomes On Line Database (GOLD) [64]. Nucleotide sequences were translated into protein sequences using the TRANSLATE web-server [65]. Position-Specific Iterated BLAST (PSI-BLAST) searches were performed against the non-redundant (nr) database at the National Center for Biotechnology Information (NCBI) http://www.ncbi.nlm.nih.gov[66] and were used to identify MBF1, TBP and Gcn4 related sequences. Sequence alignments with E-value less than 10-3 and with bit score greater than 100 were considered significant.

The analysis for functional domains was performed using Pfam protein family database http://pfam.sanger.ac.uk/[67]. Furthermore, CLUSTAL_X [54] with default settings was used to compare MBF1 and TBP sequences. The aligned sequences were inspected and adjusted manually to minimize the number of gaps and insertions. These manual adjustments were based on the sequence similarities, secondary structure prediction, and finally, fold recognition results.

The phylogenetic tree was constructed according to the Neighbor-Joining method [68] and visualized by MEGA program version 4.0 [69]. Distance analyses including 1500 bootstrap replicates were performed.

Structurally related proteins were identified by searches against known structures in the Protein Data Bank (PDB) [70], and for sequence-to-structure alignment of the deduced aMBF1 METASERVER was used [56]. METASERVER predictions were complemented with those obtained by PHYRE [57] and I-TASSER [58] for a successful fold recognition approach. The predicted 3 D structure of MBF1 was subjected to a series of tests for evaluating its internal consistency and reliability. Backbone conformation was evaluated by the inspection of the Psi/Phi Ramachandran plot obtained from PROCHECK analysis [59]. Packing quality of the 3 D model was investigated by the calculation of WHATCHECK Z-score value [60]. Finally, sequence-structure compatibility was evaluated by VERIFY-3 D [61]. All these programs were executed from the structure analysis and verification servers' web-site at UCLA http://www.doe-mbi.ucla.edu/.

## Competing interests

The authors declare that they have no competing interests.

## Authors' contributions

JMC participated in the phylogenetic and sequence analysis, carried out the molecular genetic studies and wrote the original draft of the manuscript; TP contributed to the computational analysis and 3 D modeling; AE participated in the design of the study and contributed to interpretation of the results; BS conceived the study, participated in its design and coordination and helped to draft the manuscript; all authors read, edited, and approved the final manuscript.

## Reviewers' comments

### Reviewers' report 1: Bill Martin, Institut für Botanik III, Düsseldorf University, Germany

This is a very straightforward and worthwhile manuscript investigating the ability of the archaeal bZIP protein MBF1 to complement yeast MBF deletion mutants and in domain swapping regimens. Although the complementation studies showed that only a small portion of the archaeal MBF1 (the C-terminal region) can substitute for the yeast protein in complementation studies. It would have been more exciting perhaps if the functionally more important core and N-terminal regions of the archaeal proteins from crenarchaeotes and euryarchaeotes had been functional, but they did thorough work to show that under these conditions that does not work, so that is important information for the community to know. Overall the paper was very well-prepared and is well worth publishing, basically in present form in my opinion. The only point that I stumbled upon was the six occurrences of "interestingly", which is a word that can almost always be deleted and that best occur once per paper, lest one gain the impression that everything else in the paper is not interesting (which in this case is not true). While reading I began to wonder whether the expression of archaeal specific chaperonins might help, Bogumil & Dagan (GBE 2010) have found some extensive evolutionary effects of chaperonins on protein evolution in bacteria, my hunch is that much the same exists in archaea but that it has not yet been reported. That aspect might be worth mentioning in a sentence. Otherwise this is a fine and interesting paper that will attract the interest of many microbiologists and transcription cogniscenti.

*Authors' response*: *We appreciate these constructive comments. We included the possible function of chaperonins in protein evolution in the manuscript. Although chaperonins are reported in Archaea to our knowledge their role in evolution has not been studied so far. However, it is tempting to speculate that they might be of special significance for proper protein folding at high temperature*.

*"Interestingly" has been removed from the text*.

### Reviewers' report 2: Patrick Forterre, Institut Pasteur, France

It is often claimed that Archaea use bacterial-like proteins to regulate an eukaryotic-like transcription machinery. At the cross-road between regulators and basal transcription factors, the protein MBF1, present in Archaea and Eukarya, but not in Bacteria, is an interesting model for mechanistic and evolutionary studies. In their paper, Bettina Siebers and co-workers have first used literature data and *in silico *analyses to compare the archaeal and eukaryal MBF1. In addition, they have used a MBF1 mutant from the yeast *Saccharomyces cerevisiae *to check various constructions of chimeric archaeal/eukaryal MBF1 proteins. In Eukarya, MBF1 proteins interact with TBP and regulatory proteins of the FTZ-F1 family. Whereas TBP is conserved between Archaea and Eukarya, FTZ-F1 regulators have no homologues in Archaea.

The experimental work by Siebers and co-workers shows that the archaeal MBF1 cannot complement the eukaryotic one. Chimeric proteins only work when the C-terminal domain of the eukaryal one is replaced by the archaeal one, but this domain is precisely the domain of MBF1 which is dispensable for in vivo function.

Since the central domain of MBF1 that probably interacts with TBP is conserved between Archaea and Eukarya, the lack of complementation probably came from the differences between the N-terminal domains of the Archaeal and Eukaryal MBF1 and discrete differences in the central domain. The determinants for interaction between MBF1 and FTZ-F1 are probably missing in the archaeal protein. The archaeal proteins apparently exhibit specific features that allow them to interact directly with DNA and RNA polymerase without the requirement for an additional FTZ-F1-like protein. In particular, a Zn ribbon motif present in the N-terminal part of the archaeal protein and a set of basic residues conserved in the archaeal protein could be essential for the interaction of the archaeal protein with TBP, RNA polymerase and DNA.

I think that publication of such paper in Biology Direct should be the opportunity for the authors to discuss more about the evolutionary and/or physiological aspects of their work. It seems that MBF1 in Archaea can be considered as a kind of additional basal transcription factor (dispensable since lacking in some Thaumarchaeota). What can be its role (using information from the genomic context)? How the system evolved from Archaea to eukarya or vice versa?

It would be nice if the authors could compare two scenarios, either the loss of FTZ-F1-like proteins in the archaeal lineage or their gain in the eukaryal lineage. They could possibly even suggest experiments to evolve the archaeal MBF1 protein into an archaeal-like one or vice versa.

*Authors' response*: *Considering the valuable remarks a new subsection "Origin of archaeal and eukaryotic MBF1: trends in evolution" has been included in the Results and Discussion section. We agree it would be interesting to design experiments focused on the evolution of the archaeal MBF1 protein into the archaeal like one or vice versa. Actually this was somehow the intention when we tried to work with chimeric proteins; however, it seems to be quite difficult, probably due to the acquired different functions in the course of evolution. May be directed evolution would be an alternative tool for further studies*.

A MBF1 protein is present in the recently available genome of the thaumarchaeon *Nitrososphaera gargensis*. Considering the possible position of Thaumarchaeota at the base of the archaeal tree, it should be interesting to add this protein in the phylogenetic analysis.

*Authors' response*: *MBF1 from C. N. gargensis was included in the analysis as recommended. Unfortunately, the genomic context of mbf1 in C. N. gargensis could not be analyzed, because the whole genome sequence is not available*.

### Reviewers' report 3: John van der Oost and Fabian Blombach, Wageningen University, The Netherlands

Marrero Coto and colleagues report on an interesting protein that is conserved in most Archaea and in all Eukaryotes, the Multi-protein Bridging Factor (MBF1). In order to gain insight in a function of the archaeal enzyme, complementation studies have been performed of the yeast MBF1, in which the entire yeast protein or fragments (domain swap) were substituted by its archaeal counterpart. In addition, a bioinformatics analysis has been performed. Overall the experiments have been well performed, and are well described. MBF1-TBP interaction - An extensive series of well-designed complementation experiments has been performed. Unfortunately, the outcome of the described experimental work is rather disappointing. It is concluded that the MBF1 proteins from neither *Thermoproteus *nor *Methanosarcina *are capable of functionally complementing Gcn4-dependent transcription of a yeast MBF1-mutant. In addition, it is concluded that the C-terminal extension of yeast MBF1 appears not to have an important role in the function of MBF1 as transcriptional coactivator. Obviously, more experiments are required to draw firm conclusions on the evolutionary conservation of the MBF1/TBP interaction. Based on the presented data it is impossible to conclude at what stage the complementation fails: is it because an imperfect interaction between MBF1 and TBP, between MBF1 and the bZIP regulators, or both?

*Author's response*: *We appreciate these constructive comments and recommendations. We think that the outcome of these experiments is not disappointing, but rather indicates the necessity to complement bioinformatic predictions by biological experiments. In 1999 a possible function of MBF1 in Archaea as transcription factor related to the core machinery was predicted due to the presence of the well-conserved HTH domain in Archaea and Eukaryotes and the additional Zn ribbon motif present in Archaea *[3,30]. *Liu and co-workers proposed a co-evolution of MBF1 and TBP in Eukaryotes and Archaea using bioinformatic as well as mutational analyses, suggesting the possibility of a similar role of eukaryotic and archaeal MBF1 *[30]. *Importantly it has been previously shown that archaeal TBP can be replaced by human and yeast TBP in a Methanococcus-derived cell-free transcription system *[53]*highlighting the common origin of the core transcriptional machinery. To the best of our knowledge no biological data are available that test and clarify the function of archaeal MBF1 and therefore it was a reasonable approach to test for archaeal MBF1 function in a yeast MBF1 deletion strain, especially since functional complementation by plant, human and insect MBF1 has been demonstrated *[7,21].

*The performed experiments revealed that archaeal MBF1 as well as all of the constructed chimeric proteins (except the C-terminal domain swap) are not able to complement for MBF1 function in yeast*.

*We agree that we can only speculate about the reasons why the complementation fails, but due to the presence of the well-conserved HTH domain, the absence of bZIP regulators in Archaea and the fact that eukaryotic and archaeal TBP are interchangeable in an archaeal in vitro transcription system, an impaired interaction of MBF1 with Gcn4 rather than with TBP is favourable. In the revised version of the manuscript we extended the respective discussion and are more specific about the possible explanations. In addition further experiments to analyze for yGcn4, y/aTBP, y/aMBF1 (WT and chimeric proteins) and aRNAP(subunit) interactions by yeast two hybrid studies are currently on the way in order to address this important point*.

Importantly, it is not known if all hybrid constructs are expressed in a soluble form (only shown for *T. tenax *MBF1). Although quite labor-intensive, tag-based immuno-detection and co-immuno- precipitation experiments would have been informative. The presented data do not disprove an interaction between TBP and MBF1. The failure of the archaeal HTH domains to replace the HTH of yeast MBF1 can have several reasons.

*Author's response*: *Beside T. tenax MBF1 the functional complementation of yyM and yyT confirms expression in soluble form. We agree that this is an important point and therefore we added more detailed information in the manuscript, why we choose the current approach. The aim of this study was to investigate functional complementation in yeast, which is based on the interaction of MBF1 with Tbp and Gcn4 in yeast. Therefore we avoided any tags for protein detection, which might interfere with protein-protein interaction or might influence protein-DNA interaction (e.g. by introducing a positively charged His tags)*.

*After initial negative results for aMBF1 complementation using the yeast overexpression vector (pHR98/3) we decided to express aMBF1, yMBF1 (control) as well as chimeric MBF1 s under the control of the natural yeast MBF1 promoter in order to avoid deleterious effects due to overexpression. Unfortunately the expression under control of the natural promoter and thus the low protein amounts hampered the detection of the chimeric TMBF1 proteins by the polyclonal TMBF1 antibody, although at least for yyT and yyM soluble expression was demonstrated by functional complementation*.

Being able to functionally replace yeast MBF1 by plant (Arabidopsis), insect (Bombyx mori) or human MBF1 does not mean that the same should be possible with archaeal MBF1, especially regarding the far greater phylogenetic distance.

*Author's response: We agree but a similar function of aMBF1 has been predicted previously by bioinformatic and mutational analyses *[3,30]. *A great number of publications describe different archaeal proteins that can be successfully expressed in yeast (e.g. TFBs *[50,51]*and subunits of RNAP *[52]*) and more important that are able to replace the function of eukaryotic proteins and vice versa (e.g. phophoglycerate kinase in yeast is functionally replaced by the Sulfolobus solfataricus enzyme [see Piper et al, 1996, Curr Genet]; TBP (see below) *[53]*). As mentioned above it has been shown that eukaryotic TBP is able to functionally replace archaeal TBP in a cell-free transcription assay *[53]. *Therefore the conserved function seems to be important rather than the phylogenetic distance of organisms and in our opinion it is difficult to predict a failure or success in functional replacement*.

In silico analysis of MBF1 - No conclusions are drawn from the phylogenetic analysis, and (apart from showing the conservation of the C-terminal HTH domain in the eukaryal and archaeal proteins) it does not add much to the manuscript.

*Author's response: We agree, the manuscript has been changed accordingly and the phylogeny is discussed in more detail now (see also reviewer 1 and 2)*.

As the N-terminal domains of archaeal and eukaryotic MBF1 are non-orthologous, it may be appropriate to omit them from the alignment, or at least make a clear statement on this in text and figure legend.

*Authors' response: A clear statement on the non-orthologous N-terminal domain was made in the text and in the Figure legend for clarity. Furthermore the phylogenetic tree was re-constructed based on the multiple alignment of the MBF1 sequences lacking the fragment corresponding to the Zn ribbon since the archaeal and eukaryotic N-terminal domains are non-orthologous*.

In addition, in the description of the structural model, the conservation is described of residues of the N-terminal domains of archaeal and eukaryotic MBF1 (positions 11 and 27 in *T. tenax *MBF1); this does not make sense as they are non-homologous domains. Some of the descriptions in the description of the structural models (e.g ProCheck) rather belong in the M&M section.

*Author's response: This is a misunderstanding. We modified the text for clarity. It has been mentioned in the paper that the N-terminal domain of MBF1 from Archaea has a Zn ribbon motif that is absent in the eukaryotic counterparts. The analysis of the 3D-model of T. tenax MBF1 was performed to analyze for putative van der Waals interactions between residues of the N-terminal domain and the C-terminal HTH-domain, which might contribute to fold stability of the T. tenax MBF1*.

*The description of the programs used to analyze MBF1 structural models (PROCHECK, WHATCHECK and VERIFY-3D) are declared in both Sections: in Methods and in Results and Discussion. We considered the following two sentences appropriate also for the Results and Discussion section. The exact citation, as appeared in the manuscript, is: "The structural models were analyzed by different structure validation programs including PROCHECK *[59]*for the evaluation of the Ramachandran plot quality, WHATCHECK *[60]*for the calculation of packing quality Z-score, and VERIFY-3 D *[61]*for the evaluation of sequence-structure compatibility (*Table 1*). In general, quality values obtained for the 3 D models are similar to those observed in the template structures indicating a high quality of the MBF1 low-resolution model presented in this work (*Figure 4*)"*.

The Endothelial Differentiation related factor 1 used here as template is in fact a human MBF1 ortholog; this should be mentioned in the text.

*Author's response: Modified in the text for clarity*.

Conclusions - It is proposed that, on the basis of the presented data, MBF1 most likely functions in Archaea as a specific transcriptional regulator. This is a hypothesis that could be tested experimentally, by protein-protein interaction studies (TBP, TFB, RNAP), by DNA-binding analysis, or by gene disruption.

*Author's response: We appreciate the recommendations. Moreover, the experimental investigations of the MBF1/TFB/RNAP and Gcn4 interaction (yeast-two-hybrid experiments, see above) are currently underway in the laboratory of BS at the University of Duisburg-Essen*.

In their conclusion the authors should also mention alternative functions, including on the recently suggested role of the archaeal MBF1 in ribosome biosynthesis and/or functionality.

*Author's response: The proposed role of MBF1 in ribosome biogenesis has been discussed in the manuscript for clarity; this suggested alternative function of MBF1 is now also included in the conclusions*.

## Supplementary Material

Additional file 1 **Multiple sequence alignment of archaeal and eukaryotic MBF1s**. The N-terminal domains of archaeal and eukaryotic MBF1 s are non-orthologous. The Zn ribbon motif is absent in the eukaryotic N-terminal domain. The two pairs of cysteine residues present in the N-terminal domain of archaeal MBF1 s (aMBF1) are highlighted by grey shading. Dashes indicate gaps in the amino acid sequence introduced to optimize the alignment. D112 of yMbf1, required for TBP binding in yeast, and the respective residues in eukaryotic MBF1 and aMBF1 are indicated by asterisk. The conserved motif, T(S, I)-L(V, M, F, I)-G-D(E, N, I), in the C- terminal extension of aMBF1 is indicated in bold. For the construction of the phylogenetic tree (Figure 1) the non-orthologous N-terminal domain (residues 1-37 *Ttx*MBF1) was omitted. Click here for file

Additional file 2 **Multiple sequence alignment of partial sequences of eukaryotic and archaeal MBF1 s and TBPs**. Multiple sequence alignment of partial sequences of eukaryotic and archaeal MBF1 and TBP proteins comprising analogous residues to aspartic acid at position 112 (D112, red shadow) of yMbf1 and asparagine at position 68 (Q68, blue shadow) of yTbp. A summary of compensatory amino acid changes of the interaction site of MBF1:TBP in Archaea is given in Additional File 3. Click here for file

Additional file 3 **Summary of compensatory amino acid changes of the interaction site of MBF1:TBP in Archaea**. Summary of compensatory amino acid changes of the interaction site of MBF1:TBP in Archaea based on the multiple sequence alignment shown in Additional File 2. Click here for file

Additional file 4 **List of primer sets used in this work**. List of primer sets used in this work for cloning y*MBF1*, T*MBF1*, M*MBF1 *and chimeric genes to be expressed in yeast. Underlined sequences indicate the corresponding cut-end sequence of the cloning vector used in the recombination/gap repair cloning technique. Click here for file

Additional file 5 **Plasmids generated by using the recombination/gap repair cloning technique**. Plasmids generated by using the recombination/gap repair cloning technique in order to express yMbf1, TMBF1, MMBF1 and chimera in yeast. ^a ^Plasmids generated by the use of recombination/gap repair cloning technique. ^b ^Restriction enzyme used to linearize the cloning vector. Click here for file
