# Supplementary material for: Functional analysis of archaeal MBF1 by complementation studies in yeast
Source: Biol Direct. 2011 Mar 10;6:18. doi: 10.1186/1745-6150-6-18 (PMC3062615; doi:10.1186/1745-6150-6-18)
Supplement: Additional file 1 — Multiple sequence alignment of archaeal and eukaryotic MBF1s. The N-terminal domains of archaeal and eukaryotic MBF1 s are non-orthologous. The Zn ribbon motif is absent in the eukaryotic N-terminal domain. The two pairs of cysteine residues present in the N-terminal domain of archaeal MBF1 s (aMBF1) are highlighted by grey shading. Dashes indicate gaps in the amino acid sequence introduced to optimize the alignment. D112 of yMbf1, required for TBP binding in yeast, and the respective residues in eukaryotic MBF1 and aMBF1 are indicated by asterisk. The conserved motif, T(S, I)-L(V, M, F, I)-G-D(E, N, I), in the C- terminal extension of aMBF1 is indicated in bold. For the construction of the phylogenetic tree (Figure 1) the non-orthologous N-terminal domain (residues 1-37 TtxMBF1) was omitted. [file 1745-6150-6-18-S1.PDF]

Additional file 1

```
                                                                   Zn-ribbon
S. cerevisiae         ---------------------------MSDWDTNTIIGSRARAGGSGPRANVARSQGQINAARRQGLVVS--------- 43
Y. lipolytica         --------------------------MSDDWESKTVIGSRARVGGGGPRATVAKTQAEINAAMRSGNVLS--------- 44
T. reesei             -------------------------------------------------------------------------------
D. melanogaster       ---------------------------------MSDWDSVTVLRKKAPKSSTLKTESAVNQARRQGVAVD--------- 37
B. mori               ---------------------------------MSDWDTVTILRKKPPKASALKTEQAVNAARRQGIPVD--------- 37
H. sapiens (a)        -------------------------------MAESDWDTVTVLRKKGPTAAQAKSKQAILAAQRRGEDVE--------- 39
H. sapiens (b)        -------------------------------MAESDWDTVTVLRKKGPTAAQAKSKQAILAAQRRGEDVE--------- 39
S. lycopersicum (a)   ------------------------------MSGISQDWEPVVIRKKAPTSAARKDEKAVNAARRSGAEIE--------- 40
S. lycopersicum (b)   ------------------------------MAGLSQDWEPVVIRKKAPTAAARKDEKAVNAARRAGAEIE--------- 40
S. lycopersicum (c)   -----------------------------MSGGLSQDWEPVVIRKKAPTAAARKDEKAVNAARRSGAEIE--------- 41
A. thaliana (a)       ---------------------------MAGIGPITQDWEPVVIRKKPANAAAKRDEKTVNAARRSGADIE--------- 43
A. thaliana (b)       ---------------------------MAGIGPITQDWEPVVIRKRAPNAAAKRDEKTVNAARRSGADIE--------- 43
A. thaliana (c)       -------------------------MPSRYPGAVTQDWEPVVLHKSKQKSQDLRDPKAVNAALRNGVAVQ--------- 45
Z. mays EDF1          ---------------------------MAGIGPIVQDWEPVVVPNMAPTASAMRDENAVIAARHACAEID--------- 43
T. tenax              ------------------------------MHYCDICGAPID--GEPYVIKLDNAVLHVCERCARSYGGT--------- 38
T. neutrophilus       -------------------------------MYCEICGRPIE--GEPIPIEVDKAVLYVCRSCAATYGKK--------- 37
T. pendens            ----------------------------------------------------------MCSQCAKGKTVI--------- 12
P. islandicum         -------------------------------MYCEICGRPIE--GEPIPIEVDKAVLYVCKSCAARYGKR--------- 37
P. arsenaticum        -------------------------------MYCEICGRVIE--GDPIPIEVDKAVLYVCRGCAARYGKR--------- 37
P. aerophilum         -------------------------------MYCEICGRPIE--GEPIPVEVDKAVLYVCRSCATRYGKK--------- 37
P. calidifontis       -------------------------------MYCEICGRPIE--GEPIAIELDGAVLYVCRSCAAKYGGK--------- 37
C. maquilingensis     -----------------------------MVVTCDICGREIN--GEPIVVEIDGAVLTLCQRCASRYANV--------- 39
I. hospitalis         --------------------------MKGNVLYCEMCGRPIY--GKAYRVYIEGAEMVLCESCFR-------------- 37
H. butylicus          ----------------------MTAQRRTTPLYCEMCGAPIT--GRAYRIVVEGTEMMVCERCYSRYMERSMRTGTDEP 55
S. marinus            -----------------------------MPCYCEICGREVPDERMCKTVVVDNAVLRVCPQCYRRLMKQ--------- 43
D. kamchatkensis      --------------------------MAMMSCYCEICGKEVE-KNQCRKIVIEGSILNVCPQCYNRLITQ--------- 42
A. pernix             --------------------------MKQASAYCELCGAEIR--GRPYRVSVEGVEMDLCLSCYMKLARS--------- 41
S. islandicus         -------------------------MQANSEEYCELCGSPIH--GKGITVSYEGSIITVCNSCYNRIRKH--------- 43
S. solfataricus       -------------------------MQANSEEYCELCGSQIR--GKGITVSYEGSIITVCNSCYDRIRKH--------- 43
S. acidocaldarius     -------------------------MQNQNVKYCELCGSPIK--GKGFTVSYEGSIITVCTNCFNKIKNY--------- 43
S. tokodaii           -------------------------MQSSAQKYCEMCGAPIK--GKGITVAYEGSIITVCLSCYNKIRKS--------- 43
M. sedula             ----------------------MRPMKKGVETYCEMCGNRID--GPGFSVKFEGSTITVCRSCYEKIKKH--------- 46
P. abyssi             ---------------------MGKLMAKAKPRYCELCGREIR--GEGHIIRIEGAELLVCDDCYRKYGR---------- 46
P. horikoshii         MQFALIIADRSLFNKATLKGLVGKLMAKAKPKYCELCGREIK--GEGHIIRIEGAELLVCDDCYRKYGR---------- 67
P. furiosus           -------------------------MAKAKPRYCELCGREIT--GQGHVVRIEGAELLVCDDCYRKYGR---------- 42
T. gammatolerans      ----------------------MVAMSKAKPRYCEICGAPIR--GPGHRIRLEGAEVLVCDRCYEKYGRK--------- 46
T. onnurineus         -------------------------MAKAKPKYCEICGAPIR--GPGHRIRLEGAEVLVCDRCYEKYGRK--------- 43
T. kodakarensis       -------------------------MGKAKPKYCEICGAPIR--GPGHRIRIERAEVLVCDKCYEKYGGK--------- 43
T. sibiricus          ----------------------MVLMAKAKPRICEICGAEIG--GKGHTVKIEGAELLVCHRCYEKYGRK--------- 46
T. acidophilum        -------------------------------MECEMCGKKVS-KT--TKIMIDGAVLNVCDDCAKFGTPV--------- 36
T. volcanium          -------------------------------MECEMCGKKTQ-HT--TKVMIDGAILNVCDDCAKFGTPV--------- 36
P. torridus           -------------------------------MECEMCGRNVP-QL--KRVRVSGAIMNVCPACARFGEPV--------- 36
C. K. cryptofilum     --------------------------MSGEVYVCELCGGTFY--GKPVIVDLDGYKASLCNNCARKVKKVKKKDE---- 47
M. thermautothrophicus-------------------------------MRCEICGKKIV-GK-PLKTKIDSSVMEVCRECSKFGKII--------- 37
M. smithii            -------------------------------MECEICGRQVS-DN-PKKAKIEGSVMIVCDECAKLGKIQ--------- 37
M. stadtmanae         -------------------------------MNCEICGTEIK-GQ-PYKTKIDNSLMVTCKECSRYGKVQ--------- 37
M. kandleri           ---------------------------MEERIRCEICGRVIN-GR-PKVVKVEGSELRVCEECAKFGREV--------- 41
M. acetivorans        ----------------------------MINMQCEICGAEIR-GK-PICVKIDNSELQVCQKCAPYGQPV--------- 40
M. mazei              -------------------------------MQCEICGAEIR-GK-PISVTIDNSELQVCQKCAPYGKPV--------- 37
M. barkeri            -------------------------------MQCEICGAEIR-GK-PICITIDNSELQVCQKCAPYGKPV--------- 37
M. burtoni            -------------------------------MECEICGTEIK-EK-PTDVTIDGSHLKVCSKCSQYGNAA--------- 37
M. thermophila        -------------------------MREMSDRQCEICGADIS-GS-PERIVIDGSVLEVCKSCARFGKPE--------- 43
A. fulgidus           ----------------------------MSEMNCEICGREIK-GK-GFKIVVEGSEVTVCGSCRQFGTEK--------- 40
M. marisnigri         -------------------------------MQCELCGAPIV-GP-SKTIQIEGAELCVCVRCAKHGTEV--------- 37
M. hungatei           --------------------------MMFAKMQCEMCGAEAK-GP-LKRIKIEGAELSVCNGCAKYGTEV--------- 42
M. palustris          -------------------------------MHCELCGSVIT-GPSAKRVRIEGAELEVCGQCAKYGVEV--------- 38
C. M. boonei          -------------------------------MQCEMCGETIR-GA-PKLIRVEGAELQVCAKCGKFGTEV--------- 37
M. labreanum          -----------------------MSEYIMQTEYCELCGVALS-KKGKLVQIEGAKPMRVCDKCAKLGTEV--------- 46
M. maripaludis S2     -------------------------------MQCELCGKEVK-NI--IKTRVEGVEMNVCEACAKFGMSP--------- 36
M. maripaludis S7     -------------------------------MQCELCGKEVK-DI--IKTRVEGVEMNVCEACAKFGMSP--------- 36
M. vannielii          -------------------------------MQCELCGKEVK-DI--FKTRIEGVDMNVCETCAKFGITP--------- 36
M. aeolicus           -------------------------------MQCELCGKETT-KL--LTSRIEGVEMQVCDDCAKFGTII--------- 36
M. jannaschii         -------------------------MRDSIMQMCELCGKLTD-KL--YKVIIEGSEMNVCKECAKFGKSP--------- 42
M. fervens            ------------------------------MQMCELCGKLTN-KL--YKVIIEGSEMQVCKECAKFGKSP--------- 37
M. vulcanius          ------------------------------MQMCELCGKLTE-KL--YKVVIEGSEMNVCKECAKFGKSP--------- 37
N. equitans           ------------------------------MYYCEICGDPIR--DKLYIIEIEGTKLKVCKSCSSYGKIL--------- 38
C. N. gargensis       --------------------------------------------MEKKMVIVDGTVFNVCIACSKHG------------ 23
H. utahensis          ------------------------------MVQCEMCGAETA-AP--NTVKVEGAELDVCDECADFGTEV--------- 37
H. borinquense        -----------------------------------MCGKERP-SL--TTVKVEGAELELCDDCSEFGTEV--------- 32
H. walsbyi            ------------------------------MPQCEMCGSDQS-SL--TTVKVEGAELELCGDCKEFGTEV--------- 37
H. lacusprofundi      ------------------------------MPQCEMCGADEA-SL--TTTKVEGAELELCSSCTDFGTEV--------- 37
H. marismortui        ------------------------------MVQCEMCGTEVS-SP--NRVKIEGAELDVCDECTDFGTEV--------- 37
H. mukohataei         ------------------------------MVQCEMCGKDVA-SP--NRVKIEGAELDVCDECTDFGTEV--------- 37
N. pharaonis          ------------------------------MVQCEMCGAETG-SP--KTVKIEGAELDVCDDCADFGTEV--------- 37
H. salinarum          ------------------------------MAQCEMCGTEVS-SP--KTVKVEGAEIDVCDDCAEFGTEV--------- 37
H. walsbyi (b)        --------------MPKYSTGS--GGGSGDGDSCELCGQ-ST--ATLTQANVAGAELLICEECT--PHDDAGAGPG--- 55
H. lacusprofundi (b)  --------------MAKYSTGG--GGGGDDGDACELCGR-ET--TDLQRATVAGAKLLVCSDCR--PHDDAGNAPG--- 55
H. marismortui (b)    --------------MAKYSTG---SGGDSAGGSCELCGS-DG--GDLQTANVAGATLQVCDSCAR-DHGENERTTG--- 55
H. mukohataei (b)     --------------MAKYSTG---SGSGSAGESCELCGA-SD--ADLETANVAGATLQVCADCS--QHGETSKTES--- 54
N. pharaonis (b)      --------------MAKYSTGG---VGGDESGACELCGA-ED--RSLETATVAGAELNVCDQCLKHGDGDAGQTDT--- 56
H. salinarum (b)      --------------MPKYSTGGAGSGGG--GQACELCGS-TA--DSLQDANVAGAELTVCPDCA--SHDES---AA--- 52
```

```
                                                 linker                                    ..Helix I
S. cerevisiae         ----------------VDKKYGSTNTR----GDNEGQRLTKVDRETD--------------IVKPKKLDPNVGRAISRA 88
Y. lipolytica         ----------------TDKKYASANSK----DGGDGQRLTKIDRSDD--------------IIAPPKVEASVGKAIIKG 89
T. reesei             ------------------------------AGGTEGQRLTKVDRSDD--------------IIKPKTVGKEVGKAIEQG 90
D. melanogaster       ----------------TQQKYGAGTNK----QHVTTKNTAKLDRETE--------------ELRHDKIPLDVGKLIQQG 82
B. mori               ----------------TQQKYGAGTNK----QHVTTKNTAKLDRETE--------------ELRHEKIPLDLGKLIMQG 82
H. sapiens (a)        ----------------TSKKWAAGQNK----QHSITKNTAKLDRETE--------------ELHHDRVTLEVGKVIQQG 84
H. sapiens (b)        ----------------TSKKWAAGQNK----QHSITKNTAKLDRETE--------------ELHHDRVTLEVGKVIQQG 84
S. lycopersicum (a)   ----------------TVKKSNAGSNR--AASSSTSLNTRKLDEDTE--------------NLSHEKVPTELKKAIMQA 87
S. lycopersicum (b)   ----------------TVRKATAGSNK--AASSSTTLNTRKLDEDTE--------------NLSHQKVPTELKKAIMQA 87
S. lycopersicum (c)   ----------------TIRKSTAGSNR--AASSSTTLNTRKLDEDTE--------------NLAHQKVPTELKKAIMQA 88
A. thaliana (a)       ----------------TVRKFNAGTNK--AASSGTSLNTKMLDDDTE--------------NLTHERVPTELKKAIMQA 90
A. thaliana (b)       ----------------TVRKFNAGSNK--AASSGTSLNTKKLDDDTE--------------NLSHDRVPTELKKAIMQA 90
A. thaliana (c)       ----------------TVKKFDAGSNKKGKSTAVPVINTKKLEEETE--------------PAAMDRVKAEVRLMIQKA 94
Z. mays EDF1          ----------------TMKKXNAGXNX--AASGGTSLNTKRLDDDTE--------------NLAHERVPSDLKKNLMQA 90
T. tenax              -------------------VKVESPPK--RLVQPQQRRITKRG------------------AEPRYEVVEEYAEVIKRA 78
T. neutrophilus       -------------------VIQQQAAPAVKKTP-RPKPAAPRP------------------PPPEVEIVENFGEVVKKA 78
T. pendens            ------------------GVLTFSQGAPSKPRTAVTRRRRGG--------------------EVEEIIVENYGEIIRSA 53
P. islandicum         -------------------VSPQLQQAVQKKTPPRPKSATPRL------------------QSLDVDLVENFGEVIKRA 79
P. arsenaticum        -------------------APPQTVQKKPPQSQ-KAKPPSPRL------------------PPVEVELVENYGEVIRVA 78
P. aerophilum         -------------------VLPPPPQQQQKRPVLQRPKAAPRQ------------------LPLEMELVENYADIIKRA 79
P. calidifontis       -------------------TLSKTPQPQQQKKPAKPIPQPQRS------------------RLPELELVDDFGDVIRRA 79
C. maquilingensis     ---------------KGVRVVSGVIQQTPSGQTTIKPRQRYITPVRQVKRRNEVNV----NQAERLEVIDNYGEVIKDA 99
I. hospitalis         -------------------SVKAKVAPLPKKERKAAPKPKTKK-------------------VVEYVVVEDYAERVRKA 78
H. butylicus          L----RYRLTTTRRTTQPAPREPAASAVPALRRQQARRPAALRKRET-----------SLGVVERYEVVEDYAERIRRA 119
S. marinus            -------------------GKVKEVVEPIQRTVKKTSRTQWVRARIP-----------RRLLEESYDIVEDYAERIRRA 88
D. kamchatkensis      -------------------GKARPYVEERKERQPAPKQVAKPVKP-------------R--VREEYEVVEDYARRVREA 90
A. pernix             -------------------GRAQLLREARPSRRGAARGGSGGARRP------------RRVPLDMYDLVEDYPERIREA 90
S. islandicus         -------------------AVIVKEDNKKSETQKKTTPKP------------------PKMADTELEIVADYYKIIKNA 85
S. solfataricus       -------------------AVIVKADSKKSETRKKATLKP------------------PKMENAELEIVTDYYKIIKTA 85
S. acidocaldarius     -------------------AKIVNLKKEEDKKRIKTSPN-------------------KKYTEVELDIVEEYYKIIKEA 84
S. tokodaii           -------------------AKIVNEKEIKKNEEKKKIKASTP----------------KLSTEVELEVVDDYYKLIKEA 87
M. sedula             -------------------STLVPRETKPQPAKQKRI---------------------EKAEEVELDIDEEYPRIIKEG 85
P. abyssi             -------------------KPGTFSIMPRREPTRRLTPSKPRTPTIR-----------RERPLITEDIVEDYAERVMEA 95
P. horikoshii         -------------------KPGTFSIMPRREPRRRITSSKPRVPSVR-----------REKPLITEDIVEDYAERVMEA 116
P. furiosus           -------------------KPGTFSIMPRREPTRITTSTPRKRMSPP-----------RERPLITEDIVEDYAEIVSEA 91
T. gammatolerans      -------------------KSG-FSIMPTGREPRRRPVSAPRPKREPKPY--------RERPLYTEEIVEDFAERVYRA 97
T. onnurineus         -------------------KAG-FSIMPTGRQPVRRTYSRPKPKPAPKPR--------TERPLYTEEIVEDFAERVYRA 94
T. kodakarensis       -------------------KPGTFSIMPTGRQPRRTAKPASRPKPQPRPY--------QPKPLVTEEIVEDFAERVYRA 95
T. sibiricus          -------------------KPGTWSPMPTGREPRRR--YASKPRSKPAPT--------RRKPLYTEDIVEDYADRVREA 96
T. acidophilum        -------------------IEHNKFKPVDQAIKVQLPE---RPQPRPVAIKKPA----KKVSDEDLDIVEDYAELVKNA 89
T. volcanium          -------------------IEHNNFKRVES-VKVTLPEPATRVPDTHPAKNAVK----KKISEEDLDIVEDYPEIVKNA 91
P. torridus           -------------------DEP-RKQEIKEDIKVKIPEKKIIVKTYKKPYKKYKRP--AGDDVESLDIVEDYASLIKSA 93
C. K. cryptofilum     ----------------EVTLTQKKLEVPLKKPSPSKPKEVRIPK----------------RDKGEVELVEDYGRKIREA 94
M. thermautotrophicus -------------------------REPTTPRKKSFRKPQKRSRRP---------------MDTVYEVVEDYGRIIRTE 76
M. smithii            ----------------------KAPPKPKFRKSNKPKKNKTTKQNYSK-------------DEPKEELVEDFNVKIRKA 81
M. stadtmanae         --------------------QKPQRQGNNKRNKNNQNRRTNSNRPYTRKS-----------KEEEYELVDDYEKTIKQA 85
M. kandleri           -------------------VKPRPRRETGRVQRERRPRRRPTGARRRPRGFDP--------FSEGLEVVPDYDERVREA 93
M. acetivorans        -------------------DKRTPVSRKVSPVVRAVPRTEKRPRKDF-------------FDILKDELLDNYDQIIRDA 87
M. mazei              -------------------DKRSPVSRKVSPVVRTVPRTEKRPKKDF-------------FDILKDELLDDYDHIIREA 84
M. barkeri            -------------------DKRTPVSRKVSPVVRTVTRTGNRPKKDF-------------FDILKDELLDNYDQIIRDA 84
M. burtoni            -------------------RARSPVSRKISPVSPKPIRNGPKRTTSRRDP----------FAELDDELVDEYETVMREA 87
M. thermophila        -------------------DKWSPVPRKIVPVERSFRVQKPKPRD---------------HFRDLVEVVPDYGNIIKNA 88
A. fulgidus           -------------------KPSVASQQGARRVVLKKKRGSTK-------------------IEFTEELVENFHIIIRRE 81
M. marisnigri         -------------------QQSRRRGAPQKKPGVAAPQGSRRRPR-------------DVFDLMEGELVDDYADRIRAA 84
M. hungatei           -------------------QGAVPRTSAAQAVRTSAYSGSTRPQVQRSR---------DLFDRMGGDLVEDYADRIRDA 93
M. palustris          -------------------QQPKKPALKRNVPGAKATPVVHHRR--------------DAFDMMDGEIVDDYGERIRKA 84
C. M. boonei          -------------------QQPRRTDMLRPGAPRPAPGSRAPASSAPAQRKR------DMFDYMEGEIVEDYAERVRNA 91
M. labreanum          -------------------QAPRAPVQSFGRPVITTKAKSPSQASQQANRKR------DMFDFIEGDIVEDYPQRIASA 100
M. maripaludis S2     -------------------KGYSRK-PRAVFKNETKQK-QAKRPRK------------DMFDNLK-TLVEDYGSLVKEA 81
M. maripaludis S7     -------------------KGYSRK-PKAVFKTEIKEKP-VKRPRK------------DMFDNLK-TLVEDYGSLVKEA 81
M. vannielii          -------------------KGYSRM-PKAVFNVTSDKKEKTSKKPKK-----------DMFDSLK-TIVEDYGTLIREA 83
M. aeolicus           -------------------QNYSRM-PDTKGRRSGRGTEYSSSRPKKPRK--------DLFDTLK-MVVEDYGTVIKQA 86
M. jannaschii         -------------------KTYSRLGKKTIIGKGTITTNKQVKKPIKRRR--------DIFDTLP-MLREDYGDVIREA 93
M. fervens            -------------------KTYSRLGKKPTIIGRGTSTNMQTKKPAKRRR--------DIFDTLP-MLREDYGDVIREA 88
M. vulcanius          -------------------KTYSRLGKKPTPTSGRTTT----KKPVRRKR--------DLFDSLP-VLREDYGDLIREA 84
N. equitans           ---------------------------EIIDLKKTISKEERKP---------------IVEEIIE-FVPNF-NELIKKA 73
C. N. gargensis       ------------------KPYAPAQVAAAKKKKPMAVIRQQKK---------------IGLADAT-VLTPDFARLIREA 68
H. utahensis          ------------RTQESSSASTKYSTDSSGSSSS--SSSGSASGGGGSGGSSGGGSRRDMFDEMD-ELVQDFDQRIRSA 101
H. borinquense        -----------RTESSSASGSTKYSTSSSSGSSSSSSSASSGSSSSSSSGGSTRRRR-DMFDDMD-EIAADYDDRIRQA 98
H. walsbyi            ------------RTESSSSTSTKYSTSSSNSDESSTNSTGSTTSSASQSGGSPRR---DMFDNMD-EIAADYDNRIRQA 100
H. lacusprofundi      ------------RDESTGSGGSKYSTSSSSGTSSGSSSGSSGGSSGSSGGSTRPR---DMFDNMD-EIATDYDDRIRNA 100
H. marismortui        ------------KTEETSSTSTKYSTSSSSSSSSSSSSSSSSTGGGSSGRRR------DMFDEMD-EVAQDYDDRIRKG 97
H. mukohataei         ------------TTEDSSSTSTKYSTSSSSSGSSGSSSGSSSS--SSSRPRH------DMFDEMD-ELAQDYDQRIREA 95
N. pharaonis          ------------QTQDTSSTSTKYSTSSSGSSSSGGSSGSSTSSSTGSGGGGGGGSRRDMFDEMD-EVVQDYDDRIREA 103
H. salinarum          ------------TTQSSSSTSTKYSTSSSSSQSAGSSGGSSGTNSGGRRRR-------DMFDEMD-ELAGDYDTRIRSA 96
H. walsbyi (b)        --------------SSNN-------EERSDDEPSRQKRAVQQHARMHDQA-T------SDSRHWE-REGTNYEQDQLPY 105
H. lacusprofundi (b)  GRGGSGGSR---GGSSGGSPGGASSESTGTGTESRKKEIARKQAKMYDSA-T------GDSKHWE-EGGTNYESDRLPY 123
H. marismortui (b)    NDS-----------------------SRDEQ--NRKRKAAQNAAKLQDAQ-R------ADTSHW--EDGADYDDDQLPY 100
H. mukohataei (b)     SDG-----------------------SRGDRDTDRKRRAAQNAAKMADAQ-Q------PDASHW--EDGTNYEDDQLPY 101
N. pharaonis (b)      EDR--------------------------TQERKRRKKAAQNVARLDDAR--------KVDTDWQ-ED-TEYEEDPLPY 99
H. salinarum (b)      ADA-----------------------DGDAADADRTQRAVENQAQQFDAVTR------GDSSHWE-EDGTNYETDQLPY 101
```

```
                              .......    helix II     helix    III         ..helix IV.   ..
S. cerevisiae          RT----DKKMSQKDLATKINEKPTVVNDYE----AARAIPN---QQVLSKLERALGVKLRGNNIGSPLGAPKKK----- 115
Y. lipolytica          RS----EKGLTQKELAVKINEKPQVVNDYE----SGRAQPN---QQVLSKMERVLGIKLRGKDIGLPLGPKGKK----- 152
T. reesei              RQK--FEPTMTQAELGKEIGETAATVASYE----RGTATPD---QNILSKMERVLNVKLRGANIGAPRLGPKKK----- 155
D. melanogaster        RQ----SKGLSQKDLATKICEKQQVVTDYE----AGRGIPN---NLILGKMERVLGIKLRGKERGQPIAPPGKK----- 145
B. mori                RQ----AKGMSQKDLATKICEKPQIVNDYE----AGRGIPN---NIVLGKIERAIGIKLRGKERGQPLQPPGGQK---- 146
H. sapiens (a)         RQ----SKGLTQKDLATKINEKPQVIADYE----SGRAIPN---NQVLGKIERAIGECPSTLRRVR------------- 139
H. sapiens (b)         RQ----SKGLTQKDLATKINEKPQVIADYE----SGRAIPN---NQVLGKIERAIGLKLRGKDIGKPIEKGPRAK---- 148
S. lycopersicum (a)    RQ----DKKLTQSQLAQLINEKPQIIQEYE----SGKAIPN---QQIISKLERALGAKLRGKK---------------- 139
S. lycopersicum (b)    RQ----DKKLTQSQLAQLINEKPQIIQEYE----SGKAIPN---QQIISKLERALGAKLRGKK---------------- 139
S. lycopersicum (c)    RQ----DKKLTQSQLAQLINEKPQIIQEYE----SGKAIPN---QQIISKLERALGAKLRGKK---------------- 140
A. thaliana (a)        RT----DKKLTQSQLAQIINEKPQVIQEYE----SGKAIPN---QQILSKLERALGAKLRGKK---------------- 142
A. thaliana (b)        RG----EKKLTQSQLAHLINEKPQVIQEYE----SGKAIPN---QQILSKLERALGAKLRGKK---------------- 142
A. thaliana (c)        RL----EKKMSQADLAKQINERTQVVQEYE----NGKAVPN---QAVLAKMEKVLGVKLRGKIGK-------------- 148
Z. mays EDF1           RL----DKKLTQAQLAQMINEKPQVIQEYE----SGKAIPN---QQIISKLERALGTKLREQEIAFLRVCVPEHHI--- 155
T. tenax               RE----SLGLSREALASYIGVKESVLKRIE----SGQLMPD---IELARKLEKALGVKLLEPV-QQAE--DQSGGYNRR 143
T. neutrophilus        RE----NLGLSREALAAMLGVKETVLRRIE----AGQLQPD---FALARKLEKTLGVRLLVES-VEEG-VAQTGRGAER 144
T. pendens             RE----RMGWTRDVLAAMVGEKESTIRRIE----AGQLEPT---IDLARKLEKVLKVKLIEEL-TDYG--DYSEDYGYE 118
P. islandicum          RE----NLGLSREALAAMLGIKEAVLRRIE----SGQLQPD---LALAKKLEKTLGVKLLINI-AEEG-ATSGSGRIDR 145
P. arsenaticum         RQ----NLGLSREALAAMLGVKETVLRRVE----AGQLQPD---YALAKKLEKALGVKLLVEA-KEEASGAKSGGKVER 145
P. aerophilum          RE----NLGLSRETLAAMLGVKETVLRRIE----AGQLQPD---FSLAKKLEKALGIRLLVEA-REEG-GAKAEGRLEK 145
P. calidifontis        RE----NLGLSRETLAAMLGIKETVLRRIE----AGQLQPD---LALARKIEKALGIRILVES-REET-ASSSSAKLEK 145
C. maquilingensis      RS----RLGMSRDVLASMLGIKESTLRNIE----DGKLIPD---INLARKMEKVLGIKLLVER-EEAEMEFGESGGGE- 165
I. hospitalis          RE----RLGLSRRELGMKVGEHETVIKRIE----LGRLEPD---LELARKLERVLGVELVKKVEYEESEAPKFQG--PA 144
H. butylicus           RQ----RLGLTQRELAQKVRVGENVIKRIE----AGTLVPP---IDLARRLERVLGVKLLEPVVEEELEASPRSRRDEF 187
S. marinus             RQ----RLGWTQAVLAQKVREKENVIKRIE----AGRLKPS---LELARRLEKVLKITLLEPIVEEPITSTDNE---ED 153
D. kamchatkensis       RE----RLGWTQQVLAQKVRESENIIKRIE----SGRLKPG---IDLARRLEKVLGIKLLEPVVEENVSSNHES---SE 156
A. pernix              RE----ARGWSTAVLAQKLRISETMLRRIE----SGKLKPS---LDLAKRMEKMLGVKLLEPVVDEEAYYDEDYG--RD 155
S. islandicus          RE----QLGISQQQLAQKLKVSENIVKRFE----SGKLKPT---ISQARQLEKILGIKLVTPLEN-EES---EKEFDET 149
S. solfataricus        RE----QLGISQQQLAQKLKVSENIVKRFE----SGKLKPT---ISQARQLEKILGIKLVTPLENNEES---EKEFDDT 150
S. acidocaldarius      RE----RLKMSQQQLAQALKVSENIIKRFE----SGKLKPT---IQQAKQLERILGIKLLVPIEGEEES---NP-QKDL 148
S. tokodaii            RE----RHGLSQQQLAQQLKVSENVIKRFE----SGKLKPT---IQQAKQLEKILGIKLLVPVESEEEG---E--EKDF 150
M. sedula              RE----RLHMSTKELAERMKVQENIIKRME----MGKLKPT---INEARILERILNVKLVVQVSQGKSK---SQEPDDQ 150
P. abyssi              IR----KSGLSYEELSHKVGLSVNVLRRIA----HGEYTPT---IEEAKKLERFFKIKLVERVEADFEQ--KPIIPRDY 161
P. horikoshii          IR----KSGLSYEELSHKVGLSVNLLRRIA----HGEYTPT---IEEARKLERFFKIKLVEKVEADFEQ--KPVIPRDY 182
P. furiosus            IR----KSGLSYEELSHMVGLSVNVLRRIA----HGEYTPT---IEEARKLEKFFKIKLIERVEPQFEE--KPRIPKDY 157
T. gammatolerans       IQ----RSGKSYEELSHEIGLSVNDLRAIA----HGYREPT---IKEAKKLERYFKITLIERVEEEFKE--KKTIPKDY 163
T. onnurineus          IQ----RSGKSYEELSHEIGLSMKDLRAIA----HGYREPT---IKEAKKLEKYFKITLIERVEEEVKE--KVTIPKDY 160
T. kodakarensis        IQ----RSGKSYEELSHEIGLSVNDLRAIA----HGYREPT---IKEARKLERYFKIKLIESAGEESFEE-KKTIPRDY 162
T. sibiricus           IQ----KSGLSYEELSHMVGLSTNLIRRIA----HGEYIPT---IDEAKKLERYFKIKLIERVEEEVKE--KASIPKDY 162
T. acidophilum         RE----RLAMSQADLAAKIFERKNVIASIE----RGDLMPD---LKTARKLEKILGITLVEKA---------------- 141
T. volcanium           RE----KLGMSQADLAARIFERKNVIASIE----RGDLLPD---IKTARKLEKILGITLLEKA---------------- 143
P. torridus            RE----RLSMTQEDLARKVLERKNVISNIE----RGDLLPS---IETAKKLEKVLGIKLIETEY--------------- 146
C. K. cryptofilum      RE----SLGLSIEQVAAALNIKASLLRNIE----SERVVPS---FEVARNIEKLLEVSIIQRNPERAQVTGSTPQQVYS 162
M. thermautothrophicus RE----KRDWSREDLAERINEKVSVINRIE----SERMEPD---IKLARKLERLLKIKLLEKFEADD--LEKSEGGGFR 142
M. smithii             RE----SKNLSREELGQKIYEKVSVINRIE----SGKMIPD---IRLTKKLENALNITLIENVEELD--LSKYTGNPSQ 147
M. stadtmanae          RE----KKNLTHKQLGEKIYERESVIANIE----TGKMVPD---NKIAHKLEKALHIKIIEKIESNE--REFQESRRFK 151
M. kandleri            RE----RRGWSQEDLAKKIGEKVSVIRRIE----SGKMEPD---VELARKLERVLEIELLERVSEED--TG—SVGIGSG 158
M. acetivorans         RE----AKGWSQEDLAEKIKEKASLIKKIE----RSEIVPE---DSVRKKLEHTLNIKLTERVDDAG----QEVSHMRK 151
M. mazei               RE----ARGWSQEDLAENIKEKASLIKKIE----RSEIVPE---DSVRKKLEHALNIKLTERLDDAG----QEVSHMKK 148
M. barkeri             RK----AKGWSQEDLAENIKEKVSLIKKIE----RSEIVPE---DSVRKKLEHTLNIKLTERVDESG----QEVSHLKK 148
M. burtoni             RE----KRGWTQEVLAIKIKEKASLIKKIE----RGEITPE---DSVRKKIEKALNVILMERVGKDD----WNDDRLNN 151
M. thermophila         RE----SMNLSLEDLALRIKEKASLLRKIE----REELVPE---DDVRKKLEKELKIKLTEETTEEK----LKSRGGSK 152
A. fulgidus            RE----KRGWSQEQLAKKIQEKESLIKKIE----NAEITPE---PEVVEKLEKLFNIKLREQVPEIK----IEKSK-SL 144
M. marisnigri          RE----EKEWSTLDLAHAIKEREILVKKIE----KGDLIPE---DDVRKKLEKALNIRLIDSAEDST----STGGPGRV 148
M. hungatei            RM----KLGMTQKDLALAMMERELLVKKLE----KGELIPE---DEVRKKLEKILNISLLDEGSSEP----TDLHHARM 157
M. palustris           RA----ARGMSQKDLALAVKEKEMLIKKIE----KGDLIPE---DDVRKKIEKELLIRLVDSPEDPI----EKRRADQV 148
C. M. boonei           RM----EKGISQKDLALQLMVRELLIKKIE----KGELIPE---EEVRKKLEKVLGIKLVDIVAGDD----EKKAQAKI 155
M. labreanum           RL----AKGYTQKDLAFILKMQEGDIKKFE----RGERAPT---EAERKKLEKELGIVLLDVSDDDD----KLQAGGVA 164
M. maripaludis S2      RE----KKNMTLEELSRAVGIKESLIHKIE----RNEIEPE---EKYVKILEKALGISFYE--EGDL---NYETSNEDS 144
M. maripaludis S7      RE----KKNMTLEELSRAVGIKESLIHKIE----RNEIEPE---EKYVKILEKALGISFYE--EGDY---NYEANNDES 144
M. vannielii           RE----KRNMTLEGLARTAGIKESLLHKIE----RNEIEPE---EKYVNILERELKISLYE--EGTY---NYESKDENA 146
M. aeolicus            RE----KRGWTLKELAKQIGIKESTLHKIE----RNELEPE---EKYVKRLEKELNITLYE--GSSE---EYEGGADDS 149
M. jannaschii          RE----KRGLSIEELAKKLKMKASTLQKFE----RYELEPN---EKEIKILEKELKISLTESIGEET---SYYGGRDED 158
M. fervens             RE----KRGLSIEDLAKKLKMKASTLQKFE----RYELEPN---EREIKILEKELKINLTESAGEES---PYYAGGDEE 153
M. vulcanius           RE----KMGLSIEELAKKLKMKSSTLQKFE----RYELEPN---EKEIKILEKWLKISLTENVGEDD---QFYSGESDE 149
N. equitans            RE----EKGLTQSDLAKLLHTDINTISKIE----SGDYYPS---EKLAKKIEKLLGIKIMEKKKVALS----QEKAKEE 137
C. N. gargensis        RM----KMGLTHEQLGMKMNEKAQLLRKFE----TGALKPD---ELFAKKLERYLGIKLYVSAEGSGDDNEE------- 129
H. utahensis           RE----AADMSQEELADQLNEKASLIRKLE----HGDHLPS---DDVQQKLERALDIELTESGGTDD-DADWDSGSAVG 168
H. borinquense         RE----NNGMSQEDLADSLNEKASLIRKLE----RGDILPP---DNVRKKLERKLDISLVEGGDEEE-SEWSGGSS-T 163
H. walsbyi             RE----SRSLSQEDLADSLNEKASLIRKLE----RSDILPS---DDVREKLERRLDISLVEGTDTDE--DEWSGGSS-T 165
H. lacusprofundi       RE----SRGLSQEELADQLNEKASLIRKLE----RGDTLPT---DDIQRKLESELDITLVEGESADD--ADWDSGDA-G 165
H. marismortui         RE----SQGLSQEELAKQLNEKASLIRKLE----QGNSLPS---DDVQKKLESALEISLS-AGGSAD-ETEWSGGSSDG 163
H. mukohataei          RE----ERGLTQEELAGELNLKASLIRKLE----HGDTLPS---DDVQTTLERELDISLS-AGSTDA-DEEWSGGSSSG 161
N. pharaonis           RE----AASMTQEELANSLNEKASLIRKLE----RGEVLPS---DSVQRKLERELDISLS-TGGGSD-DNDWSGGSSTG 169
H. salinarum           RE----QAGLSQEELADELNEKASVIRKLE----HGDSLPS---DDVREELEDYLGILLTESGAEDASDTDWSSGGDSA 164
H. walsbyi (b)         LV----SEYGEIAASARQAAGFT----IDE----LATELEVESGDILAVEQGRATRAGVGGSLIRELESTLDITLVDE- 161
H. lacusprofundi (b)   LV----SGYGDDVAAARQDAGLT----VEE----LAEELDVDEDDLFAVEDGRAATAGVGGSVVRALEERLGVDIVDE- 179
H. marismortui (b)     LV----SKYGERVTEARQDEGLQ----TSE----LAEELDLDDADILAVEQGRATQANVGGSTIKALEQYLDIDLVESN 156
H. mukohataei (b)      LV----SDYGSVVTEARQDAGLQ----TQE----LAEELSVDESDVLAVEQGRATQAGVGGSVVAKLEDFLDVELAE-- 157
N. pharaonis (b)       LV----SDYGTLVEQARQAEGLQ----IDE----LAREVGADEDDVVAVEQGRAARANVGGSLISALEERLDIELADT- 155
H. salinarum (b)       LV----SDYGERVVRARQDAGLQ----RSE----LAAELEIDDADVLAVEQARATKANVGGSVIAALEDFLDVQLSDD- 157
```

```
S. cerevisiae          ------------------ 151
Y. lipolytica          ------------------ 152
T. reesei              ------------------ 155
D. melanogaster        ------------------ 145
B. mori                ------------------ 146
H. sapiens (a)         ------------------ 139
H. sapiens (b)         ------------------ 148
S. lycopersicum (a)    ------------------ 139
S. lycopersicum (b)    ------------------ 139
S. lycopersicum (c)    ------------------ 140
A. thaliana (a)        ------------------ 142
A. thaliana (b)        ------------------ 142
A. thaliana (c)        ------------------ 148
Z. mays EDF1           ------------------ 155
T. tenax               ELTLGDVAELRDEE---- 157
T. neutrophilus        GLTLGEVAEIRDGGEE-- 160
T. pendens             GVTLGDIAEFRDRGDI-- 134
P. islandicum          GLTLGEVAEIRDSDEK-- 161
P. arsenaticum         GLTLGEVVEIREDGEK-- 161
P. aerophilum          GLTLGEIAEIREDGEK-- 161
P. calidifontis        GLTLGEVAEIRDEGEE-- 161
C. maquilingensis      -VTLGDVVEIRRKDKEGQ 182
I. hospitalis          ELTLGDVAVLRKE----- 157
H. butylicus           YLTLGDIAEIRED----- 200
S. marinus             YYTIGDFIKIKKKK---- 166
D. kamchatkensis       DLTIGDLIRFKRE----- 171
A. pernix              YITLGDIVVVDRDEE--- 169
S. islandicus          ELTLGDVVNIKEGKK--- 164
S. solfataricus        GLTLGDVVNIKEGKK--- 165
S. acidocaldarius      GLTLGDIVNIREGKK--- 163
S. tokodaii            ELTLGDVANIREGKK--- 165
M. sedula              TLTLGDIIRIREGKK--- 165
P. abyssi              EPTLGDIARIKIRKKKKK 179
P. horikoshii          EPTLGDIARIKVKKKKKK 200
P. furiosus            EPTLGDIARIKVKKRKK- 174
T. gammatolerans       EPTLGDIANIRIRKRKK- 180
T. onnurineus          EPTLGDIANIKIRKRKK- 177
T. kodakarensis        EPTLGDIANIRIKKRKKK 180
T. sibiricus           EPTLGDIANIRVKKKKK- 179
T. acidophilum         ------------------ 141
T. volcanium           ------------------ 143
P. torridus            ------------------ 146
C. K. cryptofilum      SVTLGEAVEVKRKRRKR- 179
M. thermautothrophicus GATIGDIARIKRG----- 155
M. smithii             GRTLGNVVKIKKR----- 160
M. stadtmanae          EATLGDIARIKRK----- 164
M. kandleri            ELTLGDVVEIRKK----- 171
M. acetivorans         DTTLGDIVKIKRK----- 164
M. mazei               DTTLGDIVKIKRK----- 161
M. barkeri             DMTLGDIVKIKRK----- 161
M. burtoni             GTTLGDIVTIKKK----- 164
M. thermophila         VLTLGDIANIRKR----- 165
A. fulgidus            VPTLGDVVVVKRKKK--- 159
M. marisnigri          TMTVGDVISFKKSRK--- 163
M. hungatei            TTTMGDVIQIKKAKK--- 172
M. palustris           KTTFGDLISIKRQP---- 162
C. M. boonei           TQTLGDLTIIRKAKK--- 170
M. labreanum           STTLGDVLQVKRK----- 177
M. maripaludis S2      EFTLGDFIKVKKRK---- 158
M. maripaludis S7      DFTLGDFIKVKNRK---- 158
M. vannielii           DFTLGDFVKIKKR----- 159
M. aeolicus            EFTLGDMIKIKRK----- 162
M. jannaschii          GFTLGDFIKIKK------ 170
M. fervens             GFTLGDFIKIKK------ 165
M. vulcanius           GFTLGDFIKIKR------ 161
N. equitans            LYSLGDIVELD------- 148
C. N. gargensis        ------------------ 129
H. utahensis           EYTLGDVVERKDS----- 181
H. borinquense         TTTLGDVVKRKD------ 175
H. walsbyi             TTTLGDVVKRKD------ 177
H. lacusprofundi       TMTLGDVVKRKD------ 177
H. marismortui         EYTLGDVVKRKD------ 175
H. mukohataei          EYTLGDVVKRKD------ 173
N. pharaonis           KTTLGDVVKRKD------ 181
H. salinarum           GLTLGDKVRRKSDDS--- 179
H. walsbyi (b)         ------------------ 171
H. lacusprofundi (b)   ------------------ 189
H. marismortui (b)     ------------------ 167
H. mukohataei (b)      ------------------ 166
N. pharaonis (b)       ------------------ 165
H. salinarum (b)       ------------------ 167
```
