# Supplementary material for: Functional analysis of archaeal MBF1 by complementation studies in yeast
Source: Biol Direct. 2011 Mar 10;6:18. doi: 10.1186/1745-6150-6-18 (PMC3062615; doi:10.1186/1745-6150-6-18)
Supplement: Additional file 2 — Multiple sequence alignment of partial sequences of eukaryotic and archaeal MBF1 s and TBPs. Multiple sequence alignment of partial sequences of eukaryotic and archaeal MBF1 and TBP proteins comprising analogous residues to aspartic acid at position 112 (D112, red shadow) of yMbf1 and asparagine at position 68 (Q68, blue shadow) of yTbp. A summary of compensatory amino acid changes of the interaction site of MBF1:TBP in Archaea is given in Additional File 3. [file 1745-6150-6-18-S2.PDF]

Additional file 2

	MBF1		TBP	Source
	helix-III			
ScMBF1	PTVVNDYEAA	sp|TBP_YEAST	PTLQNIVATVTL	Saccharomyces cerevisiae
YlMBF1	PQVVNDYESG	emb|CAG83502.1	PTLQNIVATVNL	Yarrowia lipolytica
DmMBF1a	QQVVTDYEAG	sp|TBP_DROME	PQLQNIVSTVNL	Drosophila melanogaster
BmMBF1	PQIVNDYEAG	sp|TBP_BOMMO	PQLQNIVSTVNL	Bombyx mori
hEDF1b	PQVIADYESG	sp|TBP_HUMAN	PQLQNIVSTVNL	Homo sapiens
hEDF1a	PQVIADYESG	sp|TBP_HUMAN	PQLQNIVSTVNL	Homo sapiens
AtMBF1a	PQVIQEYESG	sp|TBP1_ARATH	PTLQNIVSTVNL	Arabidopsis thaliana
AtMBF1b	PQVIQEYESG	sp|TBP2_ARATH	PTLQNIVSTVNL	Arabidopsis thaliana
ZmEDF1	PQVIQEYESG	sp|TBP1_MAIZE	PTLQNIVSTVNL	Zea mays
AtMBF1c	TQVVQEYENG	sp|TBP1_ARATH	PTLQNIVSTVNL	Arabidopsis thaliana
gb|ABL88249.1	EAVLRRIESG	sp|TBP_PYRIL	YRIENIVATVNL	Pyrobaculum islandicum DSM 4184
gb|ACB38992.1	ETVLRRIEAG	sp|TBP_THENV	YRIENIVATVNL	Thermoproteus neutrophilus V24Sta
gb|ABP49654.1	ETVLRRVEAG	gb|ABP50392.1	YRIENIVATVNL	Pyrobaculum arsenati DSM 13514
gb|AAL63025.1	ETVLRRIEAG	sp|TBP_PYRAE	YRIENIVATVNL	Pyrobaculum aerophilum str. IM2
gb|ABO09560.1	ETVLRRIEAG	sp|TBP_PYRCJ	YRIENIVATVNL	Pyrobaculum calidifontis JCM 11548
TTX_1938	ESVLKRIESG	TTX_0178	HKIENIVATVNL	Thermoproteus tenax Kra1
gb|ABW00966.1	ESTLRNIEDG	gb|ABW02033.1	YRIENIVATVNL	Caldivirga maquilinguensis IC-167
gb|ACB08362.1	ASLLRNIESE	gb|ACB08212.1	IAIQNVVSSADI	C. Korarchaeum cryptofilum OPF8
emb|CAC12077.1	KNVIASIERG	sp|TBP_THEAC	ITIENIVASTSL	Thermoplasma acidophilum DSM 1728
dbj|BAB60259.1	KNVIASIERG	sp|TBP_THEVO	ITIENIVASTSL	Thermoplasma volcanium GSS1
gb|AAT43796.1	KNVISNIERG	sp|TBP_PICTO	ITIENIVASTSL	Picrophilus torridus DSM 9790
gb|AAB85234.1	VSVINRIESE	gb|AAB86100.1	IKIQNIVASANL	Methanothermobacter thermautotrophicus
gb|ABQ86560.1	VSVINRIESG	sp|TBP_METS3	IKIENIVASASI	Methanobrevibacter smithii DSM 2375
gb|ABC57588.1	ESVIANIETG	sp|TBP_METST	IKVENIVASATL	Methanosphaera stadtmanae DSM 3091
gb|AAM01664.1	VSVIRRIESG	sp|TBP_METKA	MEIQNIVASVDL	Methanopyrus kandleri AV19
gb|AAM07611.1	ASLIKKIERS	sp|TBP1_METAC	IKIENVVASTKL	Methanosarcina acetivorans C2A
gb|AAM30701.1	ASLIKKIERS	sp|TBP1_METMA	IKIENVVASTKL	Methanosarcina mazei Go1
gb|AAZ69601.1	VSLIKKIERS	gb|AAZ70030.1	ITVENVVASTTL	Methanosarcina barkeri str. Fusaro
gb|ABE52986.1	ASLIKKIERG	gb|ABE52403.1	IKIENVVASTKL	Methanococcoides burtonii DSM 6242
gb|ABK14250.1	ASLLRKIERE	sp|TBP_METTP	INIENVVASTKL	Methanosaeta thermophila PT
gb|AAB89276.1	ESLIKKIENA	sp|TBP_ARCFU	IKIENVVASTQI	Archaeoglobus fulgidus DSM 4304
gb|ABN57234.1	EILVKKIEKG	gb|ABN57317.1	LKIENIVASAKV	Methanoculleus marisnigri JR1
gb|ABD40787.1	ELLVKKLEKG	gb|CP000254.1	YKIQNIVTSADL	Methanospirillum hungatei JF-1
gb|ACL16290.1	EMLIKKIEKG	gb|ACL16653.1	LKIENIVASAKV	Methanosphaerula palustris E1-9c
gb|ABS55980.1	ELLIKKIEKG	gb|ABS55434.1	LKIENIVASAKV	Candidatus Methanoregula boonei 6A8
gb|ABN07342.1	EGDIKKFERG	gb|ABN06621.1	LKIENIVASTKV	Methanocorpusculum labreanum Z
gb|EEJ56557.1	ASLIRKLERG	sp|C1VCY2_9EURY	IEIQNVVASTGI	Halogeometricum borinquense DSM 11551
emb|CAJ53458.1	ASLIRKLERS	emb|CAJ52639.1	IEIQNVVASTGI	Haloquadratum walsbyi DSM 16790
gb|ACM56288.1	ASLIRKLERG	gb|ACM57111.1	IEIQNVVASTGI	Halorubrum lacusprofundi ATCC 49239
gb|AAV45553.1	ASLIRKLEQG	sp|TBP_HALMA	IDIENVVASTGI	Haloarcula marismortui ATCC 43049
gb|ACV48484.1	ASLIRKLEHG	gb|ACV46852.1	INIENVVASTGI	Halomicrobium mukohataei DSM 12286
gb|ACV10547.1	ASLIRKLEHG	gb|ACV11426.1	INIENVVASTGI	Halorhabdus utahensis DSM 12940
emb|CAI49164.1	ASLIRKLERG	sp|TBP_NATPD	INIENVVASTGI	Natronomonas pharaonis DSM 2160
emb|CAP13716.1	ASVIRKLEHG	sp|TBPB_HALSA	IHIENVVASSDL	Halobacterium salinarum R1
gb|ABO36059.1	ESLIHKIERN	sp|TBP_METMP	IKVVNVVVSTQI	Methanococcus maripaludis C5
emb|CAF31202.1	ESLIHKIERN	sp|TBP_METM6	IKIVNVVVSTQI	Methanococcus maripaludis S2
gb|ABR65986.1	ESLIHKIERN	sp|TBP_METM7	IKIVNVVVSTQI	Methanococcus maripaludis C7
gb|ABR54854.1	ESLLHKIERN	sp|TBP_METVS	IKIVNVVVSTQI	Methanococcus vannielii SB
gb|ABR56610.1	ESTLHKIERN	sp|TBP_META3	IKIVNVVVSTKI	Methanococcus aeolicus Nankai-3
gb|AAB98578.1	ASTLQKFERY	sp|TBP_METJA	IKIVNVVVSTKI	Methanocaldococcus jannaschii DSM 2661
gb|ACV25177.1	ASTLQKFERY	gb|ACV24524.1	IKIVNVVVSTKI	Methanocaldococcus fervens AG86
gb|ACX73130.1	SSTLQKFERY	gb|ACX73433.1	IKIVNVVVSTKI	Methanocaldococcus vulcanius M7
gb|ABU81669.1	ETVIKRIELG	gb|ABU82168.1	IKIENIVATVSI	Ignicoccus hospitalis KIN4/I]
gb|ABM80976.1	ENVIKRIEAG	gb|ABM80782.1	AKIENIVATVSL	Hyperthermus butylicus DSM 5456
gb|ABN70014.1	ENVIKRIEAG	gb|ABN70413.1	TKIENIVATVIL	Staphylothermus marinus F1
gb|ACL11595.1	ENIIKRIESG	gb|ACL10601.1	YRVENIVATVIL	Desulfurococcus kamchatkensis 1221n
dbj|BAA81021.2	ETMLRRIESG	sp|TBP_AERPE	VKIENIVATVIL	Aeropyrum pernix K1
gb|ACR42481.1	ENIVKRFESG	gb|ACP38030.1	IQIQNIVASANL	Sulfolobus islandicus M.16.4
gb|AAK40608.1	ENIVKRFESG	sp|TBP_SULSO	VNIENIVATVTL	Sulfolobus solfataricus P2
gb|AAY80039.1	ENIIKRFESG	sp|TBP_SULAC	VNIENIVATVTL	Sulfolobus acidocaldarius DSM 639
dbj|BAB65308.1	ENVIKRFESG	sp|TBP_SULTO	VNIENIVATVTL	Sulfolobus tokodaii str. 7
gb|ABP96414.1	ENIIKRMEMG	gb|ABP95883.1	IQIQNIVASANM	Metallosphaera sedula DSM 5348
emb|CAB50187.1	VNVLRRIAHG	sp|TBP_PYRAB	LRIENIVASVDL	Pyrococcus abyssi GE5
dbj|BAA29875.1	VNLLRRIAHG	sp|TBP_PYRHO	LRIENIVASVDL	Pyrococcus horikoshii OT3
gb|AAL80636.1	VNVLRRIAHG	sp|TBP_PYRFU	LRIENIVASVDL	Pyrococcus furiosus DSM 3638
gb|ACS32956.1	VNDLRAIAHG	gb|ACS32948.1	LRIENIVASVDL	Thermococcus gammatolerans EJ3
gb|ACJ16795.1	MKDLRAIAHG	sp|TBP_THEON	LRIENIVASVDL	Thermococcus onnurineus NA1
dbj|BAD84315.1	VNDLRAIAHG	sp|TBP_PYRKO	LRIENIVASVDL	Thermococcus kodakarensis KOD1
gb|ACS89865.1	TNLIRRIAHG	sp|TBP_THESM	LRIENIVASVDL	Thermococcus sibiricus MM 739
gb|AAR38999.1	INTISKIESG	gb|NP_963333	LKLTNMVFHVRL	Nanoarchaeum equitans Kin4-M
	:		: *:* : :	
