# Supplementary material for: Functional analysis of archaeal MBF1 by complementation studies in yeast
Source: Biol Direct. 2011 Mar 10;6:18. doi: 10.1186/1745-6150-6-18 (PMC3062615; doi:10.1186/1745-6150-6-18)
Supplement: Additional file 3 — Summary of compensatory amino acid changes of the interaction site of MBF1:TBP in Archaea. Summary of compensatory amino acid changes of the interaction site of MBF1:TBP in Archaea based on the multiple sequence alignment shown in Additional File 2. [file 1745-6150-6-18-S3.PDF]

Additional file 3

Organism	Archaea equivalent residues to yTbp-Gln68	Archaea equivalent residues to yMbf1-Asp112	Reference
Yeast	Glu or Gln	Lys, Asn, Ser or Arg	[24]
Archaea	Glu	Lys, Asn, Ser, Arg, Ala	[24] and this study
	Gln	Lys, Asn or Arg	[24] and this study
	Val	Lys	This study
	Thr	Lys	This study
