# Supplementary material for: Functional analysis of archaeal MBF1 by complementation studies in yeast
Source: Biol Direct. 2011 Mar 10;6:18. doi: 10.1186/1745-6150-6-18 (PMC3062615; doi:10.1186/1745-6150-6-18)
Supplement: Additional file 4 — List of primer sets used in this work. List of primer sets used in this work for cloning yMBF1, TMBF1, MMBF1 and chimeric genes to be expressed in yeast. Underlined sequences indicate the corresponding cut-end sequence of the cloning vector used in the recombination/gap repair cloning technique. [file 1745-6150-6-18-S4.PDF]

Additional file 4

Primer Name	Sequence
yMBF1-f	aaagaattcccttctcaaacacttcaacttaa
yMBF1-r	agcggccgcaatttattaccgagtcaaatata
yv-TNt-f	taaaagctaataagctagaaaacaaaaaagttaacgagcaaaatcgtaaagaaaaatgcactactgcgacatatgc
TCt-yv-r	tgcttcattgatgacatgcagtgcgaaaagaaaggaacaaatgaaagaagacctctctattcctcatcacgtagctc
yv-MNt-f	taaaagctaataagctagaaaacaaaaaagttaacgagcaaaatcgtaaagaaaaatgcagtgcgaaatatgtggt
MCt-yv-r	tgcttcattgatgacatgcagtgcgaaaagaaaggaacaaatgaaagaagacctctttacttgcgctttattttcac
yv-yNt-f	taaaagctaataagctagaaaacaaaaaagttaacgagcaaaatcgtaaagaaaaatgtctgactgggatacaaat
yv-THTH-yv-r	gaagacctcttcatttcttctttggagctcccaaaggcgaaccgatgttgttaccctcgagcaactttacgccaag
yv-MHTH-yv-r	gaagacctcttcatttcttctttggagctcccaaaggcgaaccgatgttgttaccttcggtaagtttaatgttaag
yHTH-Tv-r	aagctctcttctgttgtatcctccagactgatcctcggcctgttgaacgggtctcaatttaacgcctaaggc
yHTH-Mv-r	ggtggtgtctttcttcatatgagagacttcctgccccgcatcatccaagcgtctcaatttaacgcctaaggc
TNt-yv-r	tctgaccctcgttgtcacccctcgtattggttgatccgtacttcttgtcaacggaaacaacgccaccataagacctagcgcaacgc
yHTH-f	gttgtttccgttgacaagaagtacgg
MNt-yv-r	tctgaccctcgttgtcacccctcgtattggttgatccgtacttcttgtcaacggaaacaaccttaccgtacggtgcacatttctggc
yHTH-yv-r	tgcttcattgatgacatgcagtgcgaaaagaaaggaacaaatgaaagaagacctcttcatctcaatttaacgcctaagg
