# Supplementary material for: Functional analysis of archaeal MBF1 by complementation studies in yeast
Source: Biol Direct. 2011 Mar 10;6:18. doi: 10.1186/1745-6150-6-18 (PMC3062615; doi:10.1186/1745-6150-6-18)
Supplement: Additional file 5 — Plasmids generated by using the recombination/gap repair cloning technique. Plasmids generated by using the recombination/gap repair cloning technique in order to express yMbf1, TMBF1, MMBF1 and chimera in yeast. a Plasmids generated by the use of recombination/gap repair cloning technique. b Restriction enzyme used to linearize the cloning vector. [file 1745-6150-6-18-S5.PDF]

Additional file 5

Plasmid name	Primer used for cloning	Cloning vector (restriction enzyme) [b]
py*MBF1*	yMBF1-f, yMBF1-r	pRS316
pT*MBF1* [a]	yv-TNt-f, TCt-yv-r	py*MBF1* (*Box*I, *Eco*81I)
pM*MBF1* [a]	yv-MNt-f, MCt-yv-r	py*MBF1* (*Box*I, *Eco*81I)
pTTy*MBF1* [a]	yv-TNt-f, yv-THTH-yv-r	py*MBF1* (*Box*I, *Eco*81I)
pyTy*MBF1* [a]	yv-yNt-f, yv-THTH-yv-r	py*MBF1* (*Box*I, *Eco*81I)
pMMy*MBF1* [a]	yv-MNt-f, yv-MHTH-yv-r	py*MBF1* (*Box*I, *Eco*81I)
pyTT*MBF1* [a]	yv-yNt-f, TCt-yv-r	py*MBF1* (*Box*I, *Eco*81I)
pyMy*MBF1* [a]	yv-yNt-f, yv-MHTH-yv-r	py*MBF1* (*Box*I, *Eco*81I)
pyMM*MBF1* [a]	yv-yNt-f, MCt-yv-r	py*MBF1* (*Box*I, *Eco*81I)
pTyy*MBF1* [a]	yv-TNt-f, TNt-yv-r	py*MBF1* (*Bgl*II)
pyyT*MBF1* [a]	yv-yNt-f, yHTH-Tv-r	pT*MBF1* (*Van*96I)
pMyy*MBF1* [a]	yv-MNt-f, MNt-yv-r	py*MBF1* (*Bgl*II)
pTyT*MBF1* [a]	yv-TNt-f, TNt-yv-r; yHTH-f, yHTH-Tv-r	pT*MBF1* (*Van*96I)
pyyM*MBF1* [a]	yv-yNt-f, yHTH-Mv-r	pM*MBF1* (*BsiW*I)
pMyM*MBF1* [a]	yv-MNt-f, MNt-yv-r; yHTH-f, yHTH-Mv-r	pM*MBF1* (*BsiW*I)
pyyΔCt*MBF1* [a]	yv-yNt-f, yHTH-yv-r	pT*MBF1* (*Van*96I)
